# Healthcare Providers’ Vaccine Perceptions, Hesitancy, and Recommendation to Patients: A Systematic Review

**DOI:** 10.3390/vaccines9070713

**Published:** 2021-07-01

**Authors:** Cheryl Lin, Jewel Mullen, Danielle Smith, Michaela Kotarba, Samantha J. Kaplan, Pikuei Tu

**Affiliations:** 1 Policy and Organizational Management Program, Duke University, Durham, NC 27705, USA; c.lin@duke.edu (C.L.); danielle.c.smith@duke.edu (D.S.); michaela.kotarba@duke.edu (M.K.); 2 Dell Medical School, The University of Texas at Austin, Austin, TX 78712, USA; jewel.mullen@austin.utexas.edu; 3 Medical Center Library and Archives, Duke University, Durham, NC 27710, USA; samantha.kaplan@duke.edu

**Keywords:** immunization, vaccine hesitancy, healthcare personnel, communication, pandemic, communicable diseases, infectious diseases, epidemiology, health behavior, health knowledge, attitudes, practice

## Abstract

Despite vaccines’ effectiveness in reducing the rate of preventable diseases, vaccine hesitancy has threatened public health and economies worldwide. Healthcare providers’ (HCP) communications and behavior strongly influence patient receptivity and uptake. The goal of this review was to examine HCP vaccine perceptions, knowledge, and reservations and how these attitudes affect their recommendations and vaccination practices. Primary research studies published by 16 September 2020 were searched in PubMed, Web of Science, Embase, CINAHL, and PsycINFO. A 14-item scale was developed for survey study and risk of bias appraisal (SSRBA). In total, 96 papers from 34 countries were included, covering 17 vaccines (HPV and influenza vaccines the most studied). Recommendation was positively associated with provider knowledge and experience, beliefs about disease risk, and perceptions of vaccine safety, necessity, and efficacy. HCP vaccination attitudes and practices varied across specialties, vaccines, and countries; demographic impact was inconclusive. Barriers included anticipation of patient/parental concerns or refusal, lacking clear guidelines, time constraints, and cost. For HPV, vaccines were more often recommended to older, female adolescents and by physicians who discussed sexual health. HCPs are vital advocates for patients and the public, but studies indicated a prevalence of provider hesitancy pertaining to inadequate knowledge, low vaccine confidence, and suboptimal uptake themselves. Improving HCP knowledge and assuring their access to information they deem trustworthy are essential to supporting HCPs‘ role as “trusted messengers” to promote vaccine acceptance.

## 1. Introduction

Vaccination is one of the most advantageous human inventions of the past two centuries, benefiting both population health and the economy [1,2]. While the effectiveness of vaccines in preventing targeted diseases has been well documented and validated [3,4], doubts about vaccine necessity or efficacy and concerns about possible adverse effects have always followed [5,6]. Despite gradual improvement over decades, U.S. vaccination rates of recommended immunizations remain suboptimal, with coverage for the childhood combined 7-vaccine series at 70.4% and for seasonal influenza at 50.4% of infants and minors and 34.2% of adults aged 18–49 years [7,8]. Similar under-vaccination is observed globally, contributing to the continuation and resurgence of infectious diseases, millions of preventable deaths, and economic burdens [9,10,11].

The World Health Organization (WHO) defined vaccine hesitancy as the context- and vaccine-specific “delay in acceptance or refusal of vaccines despite availability and quality of vaccine service” in 2014 and declared it a top global health threat in 2019 [12,13], shortly demonstrated by vaccines for the novel coronavirus SARS-CoV2 (COVID-19) that have been confronted with unprecedented debates and lack of public confidence [14,15,16]. Determinants of vaccination decision for different diseases vary across the literature. In 2019, 23% of parents in the United States expressed hesitancy toward the human papillomavirus (HPV) vaccine, and about half of them worried about its novelty and side effects [17]. A 2020 Austrian study reported 4% objection toward measles and HPV vaccines among adolescents, citing perceptions of high disease severity [18]. Another recent study on influenza vaccine found a lack of knowledge to be the primary predictor of parental hesitancy [19]. Among the key determinants related to vaccine receptivity, studies showed strong consensus on the impact of healthcare providers’ (HCPs) recommendation on patient uptake, consistent across populations and vaccines [20,21,22,23]. Earlier reviews investigated HCP beliefs and knowledge about vaccines, vaccination acceptance, awareness of guidelines, or interventions to raise HCP immunization rates [24,25,26,27,28], but few focused on how HCP viewpoints and attitudes are reflected in their practice.

HCPs are vital communicators and enablers of vaccination. This systematic review compares and synthesizes peer-reviewed studies on (a) HCP perceptions, knowledge, and reservations regarding vaccination and (b) how their vaccine attitudes manifest in their behavior, including recommendations and uptake. The study also identifies variations across HCP subgroups and different vaccines to inform research and practice, including the current COVID-19 pandemic.

## 2. Methods

### 2.1. Data Sources and Searches

A medical librarian with expertise in systematic searching composed a search utilizing a combination of subject headings and keywords to represent the concepts of vaccination, vaccine hesitancy, HCPs, patients (or parents), and recommendations. The databases MEDLINE via PubMed, Embase via Elsevier, Web of Science Core Collection Citation Indexes via Clarivate, Cumulative Index of Nursing and Allied Health Literature (CINAHL) via EBSCO, and APA PsycINFO via EBSCO were searched from inception to 16 September 2020. When possible, non-human studies, editorials, commentaries, and conference abstracts were removed (see Table S1—search strategies).

### 2.2. Study Selection

Inclusion criteria selected primary research published in English that focused on HCP opinions, knowledge, hesitancy, or practice relating to any vaccine and analyzed the influence on their communication with or recommendation to patients. Titles and abstracts were screened, followed by full-text reviews by at least two researchers via the Covidence program to determine eligibility [29]; disagreements were resolved through consensus with a third researcher. Papers that centered around patients’ perceptions, focused on vaccine development or policy, or only reported on HCP attitudes, recommendations, or barriers to providing vaccines were excluded.

### 2.3. Data Extraction and Quality Assessment

Two researchers independently extracted data into a summary table reporting the vaccine(s) studied, country of study, research design, sample size, response rate, HCP characteristics, patient population, factors influencing vaccine communication or practice, and effect statistics (Table S2).

In the absence of a gold standard for evaluating survey research for systematic review, we consulted multiple models to develop a 14-item Survey Study and Risk of Bias Appraisal (SSRBA), adapting the Circum Network’s six-survey-building-block framework (questionnaire, sampling, data collection/management/analysis, and reporting) [30], BETs’ critical appraisals for surveys and qualitative studies [31,32], and Joanna Briggs Institute’s checklist for systematic reviews [33]. Each selected paper was evaluated on sampling method and representativeness, institutional ethical approval, description of analyses, and reporting integrity (Table S3).

### 2.4. Data Synthesis and Analysis

Researchers discussed and grouped variables into emerging categories such as HCP opinions and attitudes, knowledge, and training, provider and patient characteristics, vaccine type, and external influences, noting when multiple studies supported the same findings. Associations and statistically significant values, where reported, were compiled from studies to present the direction and range of effect of each relevant factor. Findings were narratively synthesized to identify facilitators of and barriers to vaccine recommendation or provision, reported according to the Preferred Reporting Items for Systematic Reviews and Meta-Analyses (PRISMA) guidelines [34]. Analyses included impacts of HCP perceptions, knowledge of or experience with diseases or vaccines, system factors, and patients’ (or parents’) demographics and positions on vaccination. Moreover, results were synthesized in a second table to compare HCP-, vaccine-, patient-, and context-specific variables associated with recommendation; non-significant relationships were also listed in the table for a more comprehensive view of the studies reviewed.

This systematic review is registered with PROSPERO (#CRD42021225839).

## 3. Results

### 3.1. Search Results and Study Characteristics

**Figure 1:**
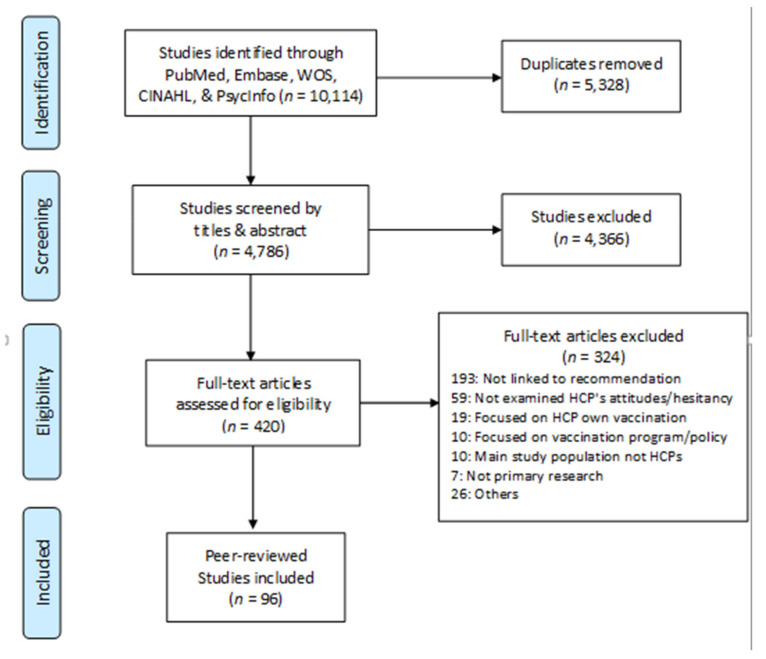
PRISMA Flow Diagram of study search and selection.

Of the 4786 search results from the 5 electronic databases, 96 studies from 34 countries were selected (Figure 1), covering 17 vaccines. HPV and influenza were the most studied. A large majority were quantitative studies conducted via email or online surveys with HCPs; four utilized qualitative interviews [35,36] and mixed methods [37,38].

A small number referenced extant models as research framework; most of these developed questionnaires based on the Health Belief Model [36,39,40,41,42,43,44,45,46,47,48] and Theory of Planned Behavior [45,49,50], and one built upon the Cognitive Model of Empowerment [51]. Studies commonly recruited HCPs from hospital staff, medical associations or conferences, and provider databases. Sample size ranged from 73 to 2962 (qualitative studies 15–34), with response rates between 6.0 and 98.6% (see Table S2—Summary Table).

Based on our SSRBA assessment, the included studies’ research design and data collection procedures were moderate- to high-quality (Table S3). Common issues included low response rates, non-random sampling, inadequate representation of the target population, and potential bias due to self-reported measures. All statistical results cited below were reported at 95% CI or *p*-value ≤ 0.05 in the original studies, otherwise denoted insignificant—most insignificant variables are recorded in Table 1.

### 3.2. HCP Attitudes, Perceptions, and Knowledge

There was ample evidence that HCPs’ attitudes toward vaccines influenced their recommendation practices across specialties, including maternity care providers [55,111], occupational physicians [126], and general practitioners (GPs) seeing older patients [75]. Pediatricians with positive attitudes of meningococcal B vaccine (4CMenB) were five times more likely to recommend it [93], as were providers who believed vaccines are effective, beneficial, and safe [36]. HCPs who believed administering vaccination and advising patients about vaccines were their responsibility had increased recommendation [71,81], discussed vaccines more often [115], and perceived greater vaccine utility [94].

Attitudes varied across vaccines and countries. Approximately 70% of Italian pediatricians deemed the HPV vaccine useful [81] and 60% considered the 4CMenB vaccine useful [93]. About 77% of French physicians reported no doubts about HPV vaccine efficacy [54], and 94% of American obstetrician-gynecologists (OB-GYNs) were confident in the vaccine’s safety and efficacy [45]. In Canada, 61% of pediatricians considered rotavirus vaccine effective [40]. HCPs in German-speaking regions in Switzerland compared to French- or Italian-speaking regions reported lower vaccine utility [94].

Measurements for attitudes and related constructs varied. The majority of the included studies employed single-item questions, summary scores [111], or preexisting scales [79], such as MoVac-flu and MovAd scales, for vaccination acceptance and engagement [51] and Multidimensional Health Locus of Control [91]. Others used Likert scales to report the level of agreement with beliefs [50,72,75,93,94,96,99,107,122,127], such as 4CMenB vaccine safety [93], normative beliefs about perinatal pertussis [122] and HPV [50], disease risk [96], and vaccine utility [75,93]. Two studies grouped the respondents based on patterns in their response to attitude-related questions [51,124].

#### 3.2.1. Vaccine and Disease Attributes

Actual recommendation, intended recommendation, or provision of vaccine were associated with viewing a vaccine as necessary (OR = 2.54) [87], useful (OR = 2.01–4.04) [39,79,81,92,93], and important [88]. Providers perceiving more “enabling than impeding factors” [35] or identifying fewer vaccination barriers were more likely to support vaccination [50,52]. Rates of recommendation decreased for HCPs with doubts about vaccine utility and necessity (OR = 0.21–0.78) [54,64,85,95]. One reason for not recommending was believing potential risks outweigh the benefits [53] (OR = 0.13) [54], as reported by 72% of non-recommenders [55].

Research frequently explored HCPs’ perceived vaccine safety and recommendation. An association existed across medical specialties for H1N1 (a type of flu virus) or 2009 pandemic influenza (OR = 2.1–10.30) [39,56,73,92] and pneumococcal conjugate vaccines (PCV) [74]. HCPs believing vaccines were safe were more likely to recommend (OR = 2.7–3.14) [42,55,56,58,59], and those expressing concerns about safety (A/OR/RR = 0.22–0.76) [42,52,55,61,62,63] or side effects (OR = 0.41–0.71) [64,65] were less likely. However, safety and side effects were most often examined by a single survey question; there was little further investigation or reporting about specific beliefs or concerns throughout the literature (e.g., identifying common or particular symptoms pertaining to certain vaccines). Physicians who adopt PCV had fewer concerns that multiple injections could cause side effects [60]. A negative relationship was observed between HCPs vaccinating their children and believing a vaccine was unsafe [58].

Likewise, vaccine efficacy was often positively associated with HCPs’ likelihood to adopt, recommend (OR = 1.38–1.61, PR = 2.1–2.6) [60,65,68,72], or intention to recommend [56,74,75] (OR = 9.07) [73]. Providers believing in vaccine efficacy were more likely to recommend vaccines against HPV [45,61,66,76,77,78], pneumococcal disease [74], pertussis [63], H1N1 [73], meningococcal group C [79], and influenza [56,68,69]. Conversely, questioning vaccine efficacy for HPV [45,77] (RR = 0.73) [61], herpes zoster (HZ) [80], and influenza [69] was negatively correlated with initiation. Multiple studies indicated insufficient information on efficacy [63,81,82] and duration of protection [83,84,85] as barriers for non-recommenders.

HCP opinion on the severity and prevalence of the disease a vaccine prevents also influenced recommendations (OR = 2.09–5.81) [8,40,42,73,75]. Increased likelihood of suggesting or administering a vaccine was observed amongst HCPs who perceived high infection risk [90,91] and high disease burden (OR = 2.75) [85], knew meningococcal group B (MenB) was spreading in the region [104], were aware diabetic patients were a vaccine priority group (OR = 6.33) [65], and believed the vaccine could reduce negative outcomes (OR = 4.90) [87]. HCPs recommending to pregnant patients believed this population was at increased risk for influenza [68] and that the tetanus, diphtheria, and pertussis (Tdap) vaccine would protect the newborn baby [88]. Given HCP exposure to infections, recommending and accepting the H1N1 vaccine was associated with believing they were at high-risk for getting (OR = 2.32) or transmitting (OR = 1.99) the virus [73]. In contrast, low recommendation rates were observed among HCPs who believed the A/H1N1 pandemic had low severity [88,89] and thought contracting varicella disease was better than vaccination [84].

#### 3.2.2. HCP Characteristics

There was less consensus on the effects of HCP demographics on recommendation. Female providers were often more likely to recommend or provide vaccines [50,60,97,98]; males were more likely to be nonadopters [99]. Some studies indicated positive vaccine behavior among older HCPs with more experience [49,73,81] (OR = 1.03) [111] or more years of practice (OR = 1.12–1.60) [65,72,100,115]. Other studies found HCPs over 50 years were less likely to recommend (OR = 0.49) [65] and recent graduates more likely (VE = 59%) [46].

Experience treating the disease a vaccine prevents facilitates recommendation [70] (OR = 1.702–7.49) [45,54,67,87,112] and reduces vaccine hesitancy [110]. Increased varicella and HPV vaccine recommendation was observed amongst HCPs caring for patients with varicella [87], cervical cancer, or other HPV-related diseases (OR = 1.46–2.3) [45,54]. Additionally, seeing a greater number of patients was correlated with recommendation [49] (OR = 20.6) [117].

Recommendation behavior differed by specialties. Pediatricians were more likely to recommend or offer vaccines [118] (AOR = 2.55) [52] than OBGYNs (AOR = 0.5) [101], family physicians (FP) (A/OR = 2.0–3.49) [46,47,84,116,127,129], and GP [46,47,118]. Pediatricians placed greater importance than FPs on guidelines (67% vs. 44%, OR = 2.6) and parental requests (61% vs. 45%, OR = 1.9) regarding varicella vaccine [84]. For the HPV vaccine, family medicine (OR = 0.13–0.24) [66], hematology/oncology (AOR = 4.69), or rheumatology (AOR = 6.55) specialization was associated with greater recommendation [103]. One study showed FPs to be less likely than gynecologists to recommend Tdap and influenza vaccines [88] while another study reported obstetricians felt less responsible than FPs to recommend (70% vs. 91%) or provide (13% vs. 86%) the influenza vaccine [111]. Furthermore, factors including being a doctor (versus a nurse) [56,73], dentist [119], pediatric resident [81], Vaccines for Children provider (AOR = 5.43) [52], or primary care provider (PCP) [98] had positive associations with accepting or recommending vaccines. Physicians without a specialty were more likely to question vaccine utility and less likely to adopt [94].

#### 3.2.3. HCP Knowledge

Knowledge was commonly measured by summary scores of correct answers to factual questions [41,71,97,101,107,109,111,113,119,126] or self-reported scales, which were more reflective of the provider’s perception of their knowledge [56,86,93]. One study found HCP self-rated knowledge of vaccine predicted their recommendation, but knowledge measured by factual questions did not [124].

Multiple studies revealed many HCPs had inadequate knowledge about vaccines or their use [81,93,101]. Such deficiency was prominent for HPV: 90% of Italian pediatricians indicated a lack of knowledge among peers [81]; only 38% of UK physicians self-reported as informed [124], and 6.5% answered all knowledge questions correctly [103]. The majority of Italian pediatricians scored low on vaccine knowledge [81], as did South African doctors [120]. Canadian HCPs showed knowledge gaps in pertussis and Tdap [38], Italian pediatricians and American PCPs in MenB disease and 4CMenB vaccine [93,104,108], and Israeli providers in childhood vaccines [107]. British GPs were less confident in their knowledge of pertussis than influenza vaccination; 59% desired further education [115]. Only 14.1% of Italian physicians were aware and knowledgeable about all recommended vaccines [96]. In addition, 70% of Thai physicians who did not recommend the influenza vaccine cited being unaware of government recommendations for pregnant women [68].

Providers knowledgeable about HPV were more likely to recommend the vaccine [41,50,101,123] (1.9–3 times) [41,101]. Likewise, providers with higher knowledge of RSV in pregnant women [86], pertussis in post-partum women [122], general influenza [91], maternal influenza [111], and 4CMenB [93] recommended the respective vaccines more frequently. Japanese providers aware of Guillain–Barre syndrome cases associated with U.S. influenza vaccination were less likely to recommend the vaccine during the 2009 H1N1 pandemic [112]. Providers with higher confidence in their knowledge elected to receive influenza vaccine more frequently [106] and recommended vaccination for influenza 1.3–3.5 times [55,106,114,115] and pertussis 6.8 times more often [114]. Reasons for not suggesting vaccines included not being confident in offering counseling [81] and inadequate vaccine training [82]. Amongst physicians, nurses, and interns, training was positively associated with recommending the influenza vaccine to diabetic patients (OR = 1.65) [65].

Having sufficient and reliable information supported vaccine advocacy [96,121] (OR = 1.7) [55]. Lacking information was correlated with not recommending [82,98] (OR = 0.40) [85], with 59.5% of non-recommenders citing this barrier [55]. Physicians who disagreed that they needed more information on the Tdap vaccine were more likely to offer it [38].

Some of these relationships were subject to change when other variables were accounted for. An Israeli study using multivariable analysis found knowledge to be associated with HCPs vaccinating their children according to the immunization program but not with their recommendation to others [113]. Self-perceived knowledge of Polish HCPs was positively associated with vaccination support, but this relationship no longer held when controlling for demographics and information source [108]. In another study, knowledge was only associated with recommendation in bivariate but not multivariate analysis, suggesting the influence of a third variable [107]. Another study found knowledge was associated with considering vaccine information reliable but not with recommendation, while considering information reliable was associated with recommendation, suggesting this perception could mediate the relationship between knowledge and advocacy [96].

#### 3.2.4. Provider Uptake

HCPs’ own vaccine uptake varied across country and vaccine, with rates for influenza ranging from 3.1% in Turkey [56] and 35.2% in the UK [91] to 78% in France [43], 91.8% in Japan [112], and almost 98% in Australia [71]. Other studies reported around 50% for Tdap vaccination [38], 45% for PCV [67], and 62.2% for H1N1 in France [43]. Even within countries, vaccination rates fluctuated—flu vaccine uptake among Italian HCPs was 60% in 2019 [44] and 22% in 2020 [96]. HCPs described as “engaged” rather than “hesitant” towards vaccination were >30 times more likely to receive vaccination [51].

Self-uptake can be a predictor of recommendation. HCPs that received or planned to receive the influenza vaccine were 2.8–8 times more likely to recommend it [39,44,56,64,67,71,91,111,128]. This relationship remained significant when adjusted for sociodemographic characteristics [106]. Regarding influenza, Tdap, Hepatitis-B, and HPV vaccines, vaccinated obstetricians were more likely to recommend vaccination to pregnant patients [95]. Providers vaccinated for hepatitis B were twice as likely to suggest it [64]. Norms and peer pressure can motivate behavior [36,108]. HCPs with vaccinated colleagues were more likely to receive vaccination [73,130]. Furthermore, providers who did not vaccinate their own children were less likely to either receive or recommend vaccines [58]. Those who did not vaccinate their daughters against HPV were 20% less likely to recommend to patients [102].

### 3.3. Patient and Contextual Factors

#### 3.3.1. Patient Characteristics

Physicians’ recommendation decisions were sometimes influenced by patient demographics [66,75,104]; 59% of FPs indicated low socioeconomic status as a barrier to prescribing non-government-funded vaccines [70]. Physicians recommended HZ vaccines more to patients ≥60 years old than to patients 50–59 years old [85], and HPV vaccines more to patients 11–12 years old than to those 13–18 years old [61,103]. HPV vaccines were less likely to be recommended by physicians seeing mostly Black adolescents (OR = 0.15) [66], and physicians who provided PCV7 had more Black patients [60].

Patient/parent or other provider refusal of vaccines [82] and perceptions of their resistance [40,107,131] impacted HCPs’ vaccination practices, such as parental concerns about vaccine safety, efficacy, or other barriers [32,73] (AOR = 0.22–0.27) [42] (IRR = 0.79–0.94) [74]. Recommendation rates were higher amongst HCPs who anticipated such concerns [77], expected patient compliance (OR = 4.907) [120], and did not believe parents would reject the vaccine [46]. Believing patients/parents lack adequate information and awareness about a vaccine [78] or would not participate in future screening [45] negatively influenced recommendation. In contrast, studies on MenC conjugate vaccine found higher recommendation toward parents who questioned the vaccine’s efficacy (OR = 3.07) [78] or doubted disease severity [79]. Other studies showed non-recommenders placed greater importance on parents’ requests for varicella vaccine than recommenders [84]. The majority of HCPs who did not recommend the HZ vaccine would still provide it at patient request [80].

Patient health was another determinant. HCPs were more likely to recommend to older patients with comorbidities [75], immunocompromised children [60], and patients with asthma [117] or an upper respiratory tract infection at an acute care visit [46] and less likely to recommend if the patient was allergic to the vaccine [94]. Higher HPV recommendation was observed for physicians seeing more patients with chronic conditions [103], and higher PCV7 recommendation for children with otitis media and who attended day care [60]. Discussing general vaccines with pregnant patients was positively associated with recommending the influenza vaccine (OR = 3.2) [55].

#### 3.3.2. System Factors

HCP vaccination behaviors were positively influenced by guidelines [50,84,86] and confidence that studies had confirmed the vaccine’s safety (AOR = 4.13) [42]. Lack of clear or official recommendation deterred suggesting [63,95,100] or administering vaccines [131]. HCPs who followed, consulted, or were aware of guidelines were more likely to advocate for vaccines (PR/OR = 1.3–3.6) [49,55,68,80,92,105,114,125], up to 22 times more for the diphtheria–tetanus–acellular pertussis (dTpa) vaccine during pregnancy [114] and over 6 times more for influenza vaccines to diabetic adults [65]. HCPs who distrusted authorities were less likely to encourage vaccination [53], and those who trusted information from institutional sources had lower hesitancy [62] and recommended more frequently [56] (OR = 1.40) [64]. Seeking information from official sources was positively associated with recommending vaccines [87], but non-recommenders often consulted news media [89], the internet, magazines, and pharmaceutical companies [129]. Believing vaccines would be accepted by vaccinators [74] (OR = 6.41) [92], vaccine providers (OR = 6.65) [39], and other professionals (OR = 7.39) [57] had positive associations. Reported barriers included lack of time [63,67,78,82,83,94], logistical difficulties [45,48,63,67,68,95,116] such as storage [85], and failure to discuss the vaccine during visits [117].

Place of practice was another frequently studied variable. Physicians working in larger [60,129], private, single-specialty (B = 0.28) [109], and solo (OR = 0.29) [72] practices, preventative rather than curative services [107], urban compared to rural locations [76,100,116], and metro versus regional areas (AOR = 0.25) [71] were more likely to recommend or adopt vaccines. Working in neighborhood or community health centers was common amongst HCPs who were reluctant to follow official organizations’ varicella vaccine recommendations [87], but another study found greater hesitancy toward the influenza vaccine in HCPs practicing in secondary (OR = 0.61) and tertiary (OR = 0.48) hospitals compared to those working in community health centers [65]. GPs at practices with alternative medicine showed greater vaccine hesitancy [110].

The considerable healthcare costs or economic burden that could result from not vaccinating against preventable diseases motivated vaccine recommendation (OR = 1.43–3.28) [39,65,74,92,94]. Vaccine cost [70,75,76,80] (OR = 0.93) [76] (r = −0.22) [40] or considering patients’ ability to pay (RR/OR = 0.57–0.76) [61,85] was an obstacle. Recommenders reported having a lower number of uninsured patients [90], and physicians with more Medicaid patients were more likely to vaccinate [60]. The Indian government’s subsidization of vaccines encouraged recommendation [132]. American physicians privately purchasing vaccines were more likely to recommend the influenza vaccine to patients with asthma (OR = 6.1) [117].

#### 3.3.3. HPV-Specific Behavior

HPV vaccines were the most studied. Recommending and providing the vaccines were more frequent for older [50,76,83,99] and female adolescents [50,99] (AOR = 6.8) [101] and patients accompanied by maternal figures rather than coming to appointments alone (AOR = 1.4) [101]. Physicians not recommending for boys reported vaccination was not as cost-effective as for girls or were unaware that it was available for boys [100]. HCPs who discussed sexual health and sexuality with patients more commonly recommended or administered the vaccine (A/OR = 2.24–2.53) [64,76,81,103,124,127]; those who regarded HPV as a public safety issue were 4.8 times more likely to recommend [97]. Reasons cited for not discussing or recommending vaccines included HCPs’ discomfort initiating conversations around sex or sexually transmitted diseases (STDs) (A/OR = 0.28–2.45) [50,66,99,103], awareness that patients were not sexually active [83], belief that discussion would increase risky sexual activity [69] (OR = 0.57) [66], and infrequent patient care visits [76,90]. Further, there were positive associations between recommending and discussing the vaccine before a patient becomes sexually active [81] or having a higher number of sexually active patients [49]. Reasons for recommending included protection from cervical cancer [81,95] and warts [95], while uncertainty about whether HPV disease actually results in cancer was negatively associated with recommendation [132]. Because HPV is an STD, HCPs worried that parents/patients would be less receptive to the vaccine [90,99] or that parents would not consider their sons’ vaccination necessary [76] and be likely to refuse it [83].

## 4. Discussion

Vaccine hesitancy and its manifestation differ among HCPs, and attitudes can vary across time and vaccines. This review detailed HCP knowledge and perceptions of vaccines in relation to recommendations. Though few included papers used the term ‘vaccine hesitancy’, as the WHO only defined it in 2014 [12], we substantiated that reluctance to recommend or provide vaccines was apparent in global research for about the past 20 years. HCPs considered various factors when making vaccination decisions, many of which were not in their control, such as system factors and patients/parents’ vaccine attitudes. Specifically, receiving encouraging information on vaccines from trustworthy medical institutions or official organizations increased HCPs’ confidence and thus likelihood to recommend vaccines, while logistical barriers such as lack of time had a negative effect. The recognition that HCPs, a group often thought of as a trustworthy source, require reliable sources of their own heightens the importance of effective provider education to facilitate their influence on patient acceptance.

This review advances insights into the perceptions of HCPs broadly, including self-perceived knowledge level, projected patients’ vaccine positions, and anticipated resistance that could affect HCP recommending practice. Perceived vaccine safety, efficacy, and utility and disease severity contributed to vaccine receptivity for HCPs, as is the case for the general public [14,133,134,135]. While side effects were commonly included in vaccine behavior research, most studies failed to specify side effects or distinguish between minor inoculation reactions versus adverse events in relation to hesitancy. Investigation into how HCPs and patients define side effects would be valuable in addressing these concerns and communicating their relative seriousness. Moreover, the included studies demonstrated providers with positive attitudes, greater knowledge, and confidence surrounding vaccines recommended more frequently, supporting evidence in other systematic reviews [24,136]. The results also indicated inadequate training, time constraints, costs, and, for the HPV vaccine, presumed or encountered parental concerns and discussion of sexual activity as deterrents to recommendation, adding to the existing literature [24,25].

The influence of HCP demographics including gender and race on vaccine behavior was inconclusive, with multiple studies reporting conflicting or insignificant relationships. Previous studies explored the impact of physician–patient congruency on interaction satisfaction and health outcomes [137,138,139]. Future investigation could determine the most relevant HCP characteristics and whether congruency can improve advocacy and uptake. Moreover, few papers examined the consistency between HCPs’ recommendation to patients and family, with some reporting divergent advice. This area lacks evidence [102,140,141] and would benefit from more research to elucidate the rationale of HCP behavior.

Two noteworthy concerns revealed by this review were that HCPs have low vaccination rates, as previous studies observed [134,142], and many have insufficient knowledge about vaccines or the diseases they prevent [143]. Educational goals should include increasing both HCPs’ competency in vaccine education and their own vaccine acceptance. Some reviews compared interventions encouraging HCP uptake, but no single program showed broad benefit across vaccines [24,144,145], necessitating further research. Lacking clear guidelines or being unaware of recommended vaccines were additional impediments in improving advocacy. Providing updated disease and vaccine information from medical associations and government agencies could build HCPs’ trust and guideline adherence, as could segmenting clinicians by specialty or patient population to increase the relevance of vaccine information provided. Furthermore, instilling the idea that vaccination is HCPs’ responsibility and offering incentives (e.g., reimbursement) could increase recommendations.

HPV vaccine studies accounted for the largest number of papers in this review. The volume or interest may reflect its unique dual role—reducing the risk of HPV infections and associated cancers. Because HPV is sexually transmitted, HCPs may find vaccine discussions uncomfortable or believe parent or patient refusal is likely. Communication training can increase vaccine initiation [27]; interventions should educate both the public and HCPs about the compound benefits to improve confidence discussing and accepting the vaccines.

Limitations of the included papers aligned with those common to survey studies, including potential biases from non-random sampling and self-reporting. Many relied on HCPs’ own perceptions of their knowledge or behavior, enabling a social desirability bias. Observation and vaccination records could be used as objective measures. Some studies lacked statistical analysis, only stating percentages or a relationship between factor and outcome, making it difficult to determine the association’s significance.

The different measures and outcome variables and inconsistent reporting limited our ability to conduct direct statistical comparisons or draw generalizable conclusions on every predictor. In addition, the amount of research which proceeds to publication is limited; studies reporting significant findings are more likely to be published and could potentially introduce bias to our review inclusion. We were mindful to list non-significant relationships in Table 1 to present different findings for comprehensive comparison. Selection bias was a possibility during our own processes, which we combatted by having multiple researchers independently select and evaluate studies and extract data for analysis. This review only included studies written in English, and this limitation was minimized by covering studies from multiple countries.

## 5. Conclusions and Future Perspectives

HCPs are a key population in the study of vaccine trust and behavior, as their recommendations influence patient acceptance. Moreover, their personal vaccination behavior affects communicable disease prevention and control in health care settings. The existing literature discussed interventions for improving the public and HCP vaccine uptakes but rarely linked HCP hesitancy to recommendation or patient vaccination. By expanding our knowledge about specific vaccine attitude–behavior associations and factors contributing to recommendation practice, this paper may guide the development of future interventions to increase HCP recommendation and their own vaccine uptake. Another strength of this study is its analysis of research across a range of countries, specialties, and vaccines. While differences existed between countries and subgroups in HCP opinions and knowledge of vaccines and diseases, our review offers evidence of consensus of their effects on recommendation.

Vaccine hesitancy has been observed for all vaccines. Only four studies in this review utilized qualitative methods. More qualitative inquiries are needed to provide insights into the nuances and formation of HCP attitudes and reservation, allowing elaboration and deeper understanding beyond predetermined quantitative scales. In addition to examining the effects of HCP factors or patient demographics, future studies on laws or mandates and recommendation guidelines in different countries or states could render lessons on how policy context drives provider practice and patient actions. Factors identified here are relevant to adults’ intention to receive COVID-19 vaccine [15,146]. With the likelihood of need for boosters and continued resistance among certain populations [14,147,148], additional research should investigate patient and HCP considerations impacting the recommendation for COVID-19 vaccine to promote herd immunity. Further examination of the roles of culture and social network in vaccination decision would better inform what interventions would prove most effective in specific communities [149]. Moreover, comparing how vaccine misinformation and confidence have developed overtime could shed light on how health crises such as pandemics and changes in the healthcare system influence vaccine opinions and actions.

## Figures and Tables

**Table 1:** Factors associated with HCP recommending or providing vaccination to patients.

Relevant Factors	More Likely to Recommend *	Less Likely to Recommend ^†^	Non-Significant ^‡^
**Vaccine and Disease Attributes**			
Weighting vaccine benefit vs. risk	perceived more benefit [35,50,52]	perceived more risk [53,54,55]	
Vaccine safety and side effect	believed safe [42,55,56,57,58,59], fewer side effect concerns [60]	with safety concern [42,52,55,61,62]; viewed safety a barrier [63]; believed vaccine could cause side effects [64,65]	safety [45,66,67,68,69,70,71], adverse events [48]
Vaccine efficacy	(likely to adopt) [60,65,68,72]; (intent to recommend) [45,56,61,63,66,68,69,73,74,75,76,77,78,79]	perceived low or unclear efficacy [45,61,63,69,77,80,81,82], waning immunity [83,84]	[69,70,71,85]
Disease severity and prevalence	[8,40,42,73,75,86]; believed vaccines reduce negative health outcomes [87]	perceived low severity [88,89]	[67]
Risk of infection	risk for patients [65,68,85,90,91,92,93]; believing they are at risk of getting/transmitting disease [73]		[65]
Vaccine utility/ necessity or importance	[7,8,15,29,40,57,87,88,94]	doubt utility/necessity [54,64,85,95]; beliefs in natural immunity [84]	[96]
**HCP Characteristics**			
Gender	female [50,60,97,98]	males more likely to be nonadopters [99]	[37,42,52,62,63,65,68,70,72,79,83,90,100,101,102,103,104,105,106,107,108,109,110]
Race and ethnicity	Indian doctors in Malaysia [100]		[52,66,86,109]
Age and experience	older or more experienced [49,73,81,111]	older [65]	Age [42,63,72,90,97,100,101,104,105,106,108,109,112]; experience [54,63,87,113,114]
Number of years practicing	greater number [65,72,100,115]; recent graduates [46]		year of graduation [47]; time practicing [56,71,82,116]
History treating the disease	[45,54,67,70,87,110,112]		
Number of patients seen	greater number [49,117]		[72]
Specialty	pediatricians [39,50,54,57,58,59,101,118], FP [66], hematology/oncology or rheumatology [103], PCP [98], doctor (vs. Nurse) [56,73], dentist (vs. Hygienist) [119], pediatric resident [81], Vaccines for Children doctors [52]	FP [88], obstetrician [111], physicians w/out a specialty [94]	[47,68,71,85,97,115,116]
Level of training	training on disease [65]; adopting new technology [45,77]	inadequate training [82]; insufficient info to counsel [81]	[69,70]
Information	adequate/reliable [38,42,55,70,96,120,121]	lack information [30,44,55,82,85,98]	needing more info [72]; felt poorly informed [78]
Believing vaccination was their responsibility	(recommending) [71,81], (discussing) [115]		
Knowledge of vaccine and/or disease	[41,50,86,91,93,101,111,122,123]; confidence in vaccine study [42]	aware Guillain–Barre disease related to influenza vaccine [112]	[56,78,97,99,103,108,109,124]
Knowledge of guidelines	[65,68,86,93,105,114,125]		[126]
Confidence in knowledge	[51,55,64,106,114,115]		confidence in general [127]
Attitudes towards vaccines	positive attitudes [55,75,93,111,126]; perceive vaccine acceptance by others [39,57,74,92]; believing vaccines reduce parents losing work time [87]		
Own uptake of a vaccine	[39,44,56,67,71,91,95,106,111,128]	not vaccinating own child [54,58,102]	[67,112,129]
Perceived norm or expectation	peer pressure [36,108]; having vaccinated colleagues [73,130]		
**Patient Characteristics**			
Demographics	age [66,75,104]; older [85]; seeing more Black patients [60]	low socioeconomic status [70]; seeing mostly non-Hispanic white [66]	age [101]; race [103]
Political Views	non-conservative [101]		
Patient/parent behavior, views, or related factors	HCP not believing parents would reject [46], anticipating concerns [77], expecting compliance [120]; patients not understanding disease severity [59]; parents not believing in efficacy [78]	expressed refusal or concerns [32,40,42,73,74,82,107]; believing patients/parents lack adequate info [78], would not complete future screening [45]; HCP placing emphasis on parental request [84]	
Patient condition	older, with comorbidities [75]; children with asthma [117]; immunocompromised [60]; upper respiratory tract infection [46]; chronic medical conditions [103]; attending day care [60]; pregnant patients [55]	patient allergic to something in vaccine [94]	
**System Factors**			
Recommendations and guidelines	having guidelines [50,84]; following guidelines [49,55,68,80,92,105,114]	lacking guidelines [63,95,100,131]	
Trust in authorities or information sources	trust [56,62,64]	lack of trust [53]	[58]
Sources of information	official sources [87];	media [89]; internet, magazines, pharmaceutical companies [129]	[89,108,125]
Barriers to recommendation		lack of time [63,67,78,82,83,94] logistical difficulties [45,48,63,67,68,95,116] failure to discuss vaccine [117]	(not having) time to discuss [127]
Place of practice	private [104,109]; larger [60,129]; solo practice [72]; preventative [107]; urban [76,100,116]; metro [71]	neighborhood or community health centers [87]; secondary/tertiary hospital [65]; practices where alternative medicine used [110]	location [42,63,82,83,100,103,104,105,131]; practice type [65,68,79,103,105,114,131]; private practice [128]
Cost	cost of disease [39,65,74,92,94]; free vaccine [132]; having privately purchased vaccines [117]	cost of vaccine [27,40,76]; patient ability to pay or financial burden [61,85]; high cost or non-funded [70]	vaccine cost [66,70,78,85,127]; reimbursed [42,66]; patient payment [132]; affordability [67]; too costly to store [87]
Patient insurance	seeing fewer uninsured patients [90]; more Medicaid patients [60]		number of Medicaid patients [104,117]; insurance status [103]
**HPV Specific**			
Patient demographics	older [50,76,83,99]; female [50,99,101]	infrequent visits [76,90]	
Factors regarding sexual health or sexuality	discussed sexuality & sexual health [76,81,103,124,127]; comfortable discussing [64]; discussion prior to sexual activity [81]; having more sexually active patients [49]	feeling uncomfortable discussing [50,66,99,103]; aware patient not sexually active [83]; believing discussion would increase sexual activity [66,69]	discussing sex [52]; believing discussion would increase sexual activity [78,132]; comfortable discussing sexuality & sexual health [99,129]
Believing HPV is a public safety issue	[97]		
Resulting diseases	believing vaccine prevents cervical cancer and warts [81,95]	uncertain if HPV results in cancer [132]	
Patient and parental attitudes	to patient accompanied by mother to appointment [101]	believing they will be less receptive [90,99], refuse [83], not vaccinate their sons [76]	[77,78]

* Variables in this column were positively associated with either recommendation or vaccine adoption. ^†^ Variables in this column were associated with less likelihood of recommendation or adoption or cited as a barrier. ^‡^ Factors are denoted insignificant if *p*-value was reported ≥0.05 or specified by the authors as non-significant.

## Data Availability

Not applicable.

## Supplementary Materials

The following are available online at https://www.mdpi.com/article/10.3390/vaccines9070713/s1, Table S1: Literature Search Strategies, Table S2: Summary Table of Included Studies, Table S3: 14-item Survey Study and Risk of Bias Appraisal (SSRBA)*.
