# Supplementary material for: Healthcare Providers’ Vaccine Perceptions, Hesitancy, and Recommendation to Patients: A Systematic Review
Source: Vaccines (Basel). 2021 Jul 1;9(7):713. doi: 10.3390/vaccines9070713 (PMC8310254; doi:10.3390/vaccines9070713)
Supplement: Supplementary file 1 [file vaccines-09-00713-s001.zip › vaccines-1219952-supplementary.pdf]

vaccines

MDPI

Supplementary 1. Literature Search Strategies

Database: MEDLINE via PubMed Search Date: 9-16-2020; searcher: SJK

Set #		Results
1	"Vaccines"[Mesh] OR "Immunization"[Mesh] OR "Immunization Schedule"[Mesh] OR "Vaccination"[Mesh] OR vaccine[tiab] OR vaccines[tiab] OR vaccination[tiab] OR vaccinate[tiab] OR vaccinated[tiab] OR immunization[tiab] OR immunizations[tiab] OR immunisation[tiab] OR immunisations[tiab] OR immunise[tiab] OR immunises[tiab] OR immunised[tiab]	460,171
2	"Anti-Vaccination Movement"[Mesh] OR "Vaccination Refusal"[Mesh] OR "Health Knowledge, Attitudes, Practice"[Mesh] OR attitudes[tiab] OR attitude[tiab] OR avoidance[tiab] OR avoids[tiab] OR avoid[tiab] OR avoided[tiab] OR choices[tiab] OR choice[tiab] OR choose[tiab] OR choosing[tiab] OR concern[tiab] OR concerns[tiab] OR concerned[tiab] OR confidence[tiab] OR confident[tiab] OR decision[tiab] OR decisions[tiab] OR decide[tiab] OR decided[tiab] OR deciding[tiab] OR decides[tiab] OR delay[tiab] OR delays[tiab] OR delaying[tiab] OR delayed[tiab] OR distrust[tiab] OR distrusting[tiab] OR distrusted[tiab] OR doubt[tiab] OR doubts[tiab] OR doubting[tiab] OR doubted[tiab] OR fear[tiab] OR fears[tiab] OR fearing[tiab] OR hesitation[tiab] OR hesitates[tiab] OR hesitate[tiab] OR hesitated[tiab] OR hesitancy[tiab] OR knowledge[tiab] OR misconception[tiab] OR misinformation[tiab] OR misinformed[tiab] OR mistrust[tiab] OR opposition[tiab] OR oppose[tiab] OR opposing[tiab] OR opposes[tiab] OR opposed[tiab] OR perceived[tiab] OR perception[tiab] OR perceptions[tiab] OR refusal[tiab] OR refuses[tiab] OR refused[tiab] OR refusals[tiab] OR refusing[tiab] OR refuse[tiab] OR rejection[tiab] OR rejects[tiab] OR reject[tiab] OR rejected[tiab] OR trust[tiab] OR trusts[tiab] OR trusted[tiab] OR trusting[tiab] OR uptake[tiab]	3,900,232
3	"Health Personnel"[Mesh] OR "Attitude of Health Personnel"[Mesh] OR "healthcare worker"[tiab] OR "healthcare workers"[tiab] OR "healthcare provider"[tiab] OR "healthcare providers"[tiab] OR "healthcare professionals"[tiab] OR "healthcare professional"[tiab] OR practitioner[tiab] OR practitioners[tiab] OR physician[tiab] OR physicians[tiab] OR doctor[tiab] OR doctors[tiab] OR clinician[tiab] OR clinicians[tiab] OR HCP[tiab] OR HCPs[tiab] OR nurse[tiab] OR nurses[tiab] OR "physician assistant"[tiab] OR "physician's assistant"[tiab] OR "physician assistants"[tiab] OR "physician's assistants"[tiab] OR pharmacists[tiab] OR pharmacist[tiab]	1,448,209
4	"Adult"[Mesh] OR "Pregnant Women"[Mesh] OR "Parents"[Mesh] OR adult[tiab] OR adults[tiab] OR parenting[tiab] OR parent[tiab] OR parents[tiab] OR mother[tiab] OR father[tiab] OR mothers[tiab] OR fathers[tiab] OR parenthood[tiab] OR parental[tiab] OR Patient[tiab] OR patients[tiab] OR ((pregnant[tiab] OR pregnancy[tiab]) AND (woman[tiab] OR women[tiab] OR person[tiab] OR persons[tiab]))	11,274,854
5	recommend[tiab] OR recommends[tiab] OR recommendation[tiab] OR recommendations[tiab] OR recommending[tiab] OR recommended[tiab] OR communication[tiab] OR communications[tiab] OR communicated[tiab] OR communicate[tiab] OR communicating[tiab] OR communicates[tiab]	950,717
	1 AND 2 AND 3 AND 4 AND 5	2,868
	NOT (animals[mh] NOT humans[mh])<br>NOT (Editorial[ptyp] OR Comment[ptyp])	2,848

Database: Embase via Elsevier

Set #		Results
1	'vaccine'/exp OR 'immunization'/exp OR 'vaccination'/exp OR vaccine:ti,ab OR vaccines:ti,ab OR vaccination:ti,ab OR vaccinate:ti,ab OR vaccinated:ti,ab OR immunization:ti,ab OR immunizations:ti,ab OR immunisation:ti,ab OR immunisations:ti,ab OR immunise:ti,ab OR immunises:ti,ab OR immunised:ti,ab	609,205
2	'anti-vaccination movement'/exp OR 'vaccination refusal'/exp OR 'attitude to health'/exp OR attitudes:ti,ab OR attitude:ti,ab OR avoidance:ti,ab OR avoids:ti,ab OR avoid:ti,ab OR avoided:ti,ab OR choices:ti,ab OR choice:ti,ab OR choose:ti,ab OR choosing:ti,ab OR concern:ti,ab OR concerns:ti,ab OR concerned:ti,ab OR confidence:ti,ab OR confident:ti,ab OR decision:ti,ab OR decisions:ti,ab OR decide:ti,ab OR decided:ti,ab OR deciding:ti,ab OR decides:ti,ab OR delay:ti,ab OR delays:ti,ab OR delaying:ti,ab OR delayed:ti,ab OR distrust:ti,ab OR distrusting:ti,ab OR distrusted:ti,ab OR doubt:ti,ab OR doubts:ti,ab OR doubting:ti,ab OR doubted:ti,ab OR fear:ti,ab OR fears:ti,ab OR fearing:ti,ab OR hesitation:ti,ab OR hesitates:ti,ab OR hesitate:ti,ab OR hesitated:ti,ab OR hesitancy:ti,ab OR knowledge:ti,ab OR misconception:ti,ab OR misinformation:ti,ab OR misinformed:ti,ab OR mistrust:ti,ab OR opposition:ti,ab OR oppose:ti,ab OR opposing:ti,ab OR opposes:ti,ab OR opposed:ti,ab OR perceived:ti,ab OR perception:ti,ab OR perceptions:ti,ab OR refusal:ti,ab OR refuses:ti,ab OR refused:ti,ab OR refusals:ti,ab OR refusing:ti,ab OR refuse:ti,ab OR rejection:ti,ab OR rejects:ti,ab OR reject:ti,ab OR rejected:ti,ab OR trust:ti,ab OR trusts:ti,ab OR trusted:ti,ab OR trusting:ti,ab OR uptake:ti,ab	5,214,869
3	'health care personnel'/exp OR "healthcare worker":ti,ab OR "healthcare workers":ti,ab OR "healthcare provider":ti,ab OR "healthcare providers":ti,ab OR "healthcare professionals":ti,ab OR "healthcare professional":ti,ab OR practitioner:ti,ab OR practitioners:ti,ab OR physician:ti,ab OR physicians:ti,ab OR doctor:ti,ab OR doctors:ti,ab OR clinician:ti,ab OR clinicians:ti,ab OR HCP:ti,ab OR HCPs:ti,ab OR nurse:ti,ab OR nurses:ti,ab OR "physician assistant":ti,ab OR "physician s assistant":ti,ab OR "physician assistants":ti,ab OR "physician s assistants":ti,ab OR pharmacists:ti,ab OR pharmacist:ti,ab	2,499,500
4	'adult'/exp OR 'pregnant woman'/exp OR 'parent'/exp OR adult:ti,ab OR adults:ti,ab OR parenting:ti,ab OR parent:ti,ab OR parents:ti,ab OR mother:ti,ab OR father:ti,ab OR mothers:ti,ab OR fathers:ti,ab OR parenthood:ti,ab	15,236,613

	OR parental:ti,ab OR Patient:ti,ab OR patients:ti,ab OR ((pregnant:ti,ab OR pregnancy:ti,ab) AND (woman:ti,ab OR women:ti,ab OR person:ti,ab OR persons:ti,ab))	
5	recommend:ti,ab OR recommends:ti,ab OR recommendation:ti,ab OR recommendations:ti,ab OR recommending:ti,ab OR recommended:ti,ab OR communication:ti,ab OR communications:ti,ab OR communicated:ti,ab OR communicate:ti,ab OR communicating:ti,ab OR communicates:ti,ab	1,354,555
	1 AND 2 AND 3 AND 4 AND 5	4,720
	AND [humans]/lim	4,484
	NOT 'conference abstract'/it	3,385

Database: Web of Science Core Collection Citation Indexes via Clarivate

Set #		Results
1	TS=(vaccine OR vaccines OR vaccination OR vaccinate OR vaccinated OR immunization OR immunizations OR immunisation OR immunisations OR immunise OR immunises OR immunized)	401,219
2	TS=(attitudes OR attitude OR avoidance OR avoids OR avoid OR avoided OR choices OR choice OR choose OR choosing OR concern OR concerns OR concerned OR confidence OR confident OR decision OR decisions OR decide OR decided OR deciding OR decides OR delay OR delays OR delaying OR delayed OR distrust OR distrusting OR distrusted OR doubt OR doubts OR doubting OR doubted OR fear OR fears OR fearing OR hesitation OR hesitates OR hesitate OR hesitated OR hesitancy OR knowledge OR misconception OR misinformation OR misinformed OR mistrust OR opposition OR oppose OR opposing OR opposes OR opposed OR perceived OR perception OR perceptions OR refusal OR refuses OR refused OR refusals OR refusing OR refuse OR rejection OR rejects OR reject OR rejected OR trust OR trusts OR trusted OR trusting OR uptake)	8,218,587
3	TS=("healthcare worker" OR "healthcare workers" OR "healthcare provider" OR "healthcare providers" OR "healthcare professionals" OR "healthcare professional" OR practitioner OR practitioners OR physician OR physicians OR doctor OR doctors OR clinician OR clinicians OR HCP OR HCPs OR nurse OR nurses OR "physician assistant" OR "physician's assistant" OR "physician assistants" OR "physician's assistants" OR pharmacists OR pharmacist)	1,116,843
4	TS=(adult OR adults OR parenting OR parent OR parents OR mother OR father OR mothers OR fathers OR parenthood OR parental OR Patient OR patients OR ((pregnant OR pregnancy) AND (woman OR women OR person OR persons)))	8,211,035
5	TS=(recommend OR recommends OR recommendation OR recommendations OR recommending OR recommended OR communication OR communications OR communicated OR communicate OR communicating OR communicates)	1,963,863
	1 AND 2 AND 3 AND 4 AND 5	2,474

Database: CINAHL (Cumulative Index of Nursing and Allied Health Literature via EBSCO)

Set #		Results
1	(MH "Vaccines+") OR (MH "Immunization+") OR TI vaccine OR AB vaccine OR TI vaccines OR AB vaccines OR TI vaccination OR AB vaccination OR TI vaccinate OR AB vaccinate OR TI vaccinated OR AB vaccinated OR TI immunization OR AB immunization OR TI immunizations OR AB immunizations OR TI immunisation OR AB immunisation OR TI immunisations OR AB immunisations OR TI immunise OR AB immunise OR TI immunises OR AB immunises OR TI immunised OR AB immunised	76,762
2	(MH "Anti-Vaccination Movement") OR (MM "Treatment Refusal") OR TI attitudes OR AB attitudes OR TI attitude OR AB attitude OR TI avoidance OR AB avoidance OR TI avoids OR AB avoids OR TI avoid OR AB avoid OR TI avoided OR AB avoided OR TI choices OR AB choices OR TI choice OR AB choice OR TI choose OR AB choose OR TI choosing OR AB choosing OR TI concern OR AB concern OR TI concerns OR AB concerns OR TI concerned OR AB concerned OR TI confidence OR AB confidence OR TI confident OR AB confident OR TI decision OR AB decision OR TI decisions OR AB decisions OR TI decide OR AB decide OR TI decided OR AB decided OR TI deciding OR AB deciding OR TI decides OR AB decides OR TI delay OR AB delay OR TI delays OR AB delays OR TI delaying OR AB delaying OR TI delayed OR AB delayed OR TI distrust OR AB distrust OR TI distrusting OR AB distrusting OR TI distrusted OR AB distrusted OR TI doubt OR AB doubt OR TI doubts OR AB doubts OR TI doubting OR AB doubting OR TI doubted OR AB doubted OR TI fear OR AB fear OR TI fears OR AB fears OR TI fearing OR AB fearing OR TI hesitation OR AB hesitation OR TI hesitates OR AB hesitates OR TI hesitate OR AB hesitate OR TI hesitated OR AB hesitated OR TI hesitancy OR AB hesitancy OR TI knowledge OR AB knowledge OR TI misconception OR AB misconception OR TI misinformation OR AB misinformation OR TI misinformed OR AB misinformed OR TI mistrust OR AB mistrust OR TI opposition OR AB opposition OR TI oppose OR AB oppose OR TI opposing OR AB opposing OR TI opposes OR AB opposes OR TI opposed OR AB opposed OR TI perceived OR AB perceived OR TI perception OR AB perception OR TI perceptions OR AB perceptions OR TI refusal OR AB refusal OR TI refuses OR AB refuses OR TI refused OR AB refused OR TI refusals OR AB refusals OR TI refusing OR AB refusing OR TI refuse OR AB refuse OR TI rejection OR AB rejection OR TI rejects OR AB rejects OR TI reject OR AB reject OR TI rejected OR AB rejected OR TI trust OR AB trust OR TI trusts OR AB trusts OR TI trusted OR AB trusted OR TI trusting OR AB trusting OR TI uptake OR AB uptake	1,097,114
3	(MH "Health Personnel+") OR (MH "Attitude of Health Personnel+") OR TI "healthcare worker" OR AB "healthcare worker" OR TI "healthcare workers" OR AB "healthcare workers" OR TI "healthcare provider" OR AB "healthcare provider" OR TI "healthcare providers" OR AB "healthcare providers" OR TI "healthcare professionals" OR AB "healthcare professionals" OR TI "healthcare professional" OR AB "healthcare professional" OR TI	1,112,322

	practitioner OR AB practitioner OR TI practitioners OR AB practitioners OR TI physician OR AB physician OR TI physicians OR AB physicians OR TI doctor OR AB doctor OR TI doctors OR AB doctors OR TI clinician OR AB clinician OR TI clinicians OR AB clinicians OR TI HCP OR AB HCP OR TI HCPs OR AB HCPs OR TI nurse OR AB nurse OR TI nurses OR AB nurses OR TI "physician assistant" OR AB "physician assistant" OR TI "physician's assistant" OR AB "physician's assistant" OR TI "physician assistants" OR AB "physician assistants" OR TI "physician's assistants" OR AB "physician's assistants" OR TI pharmacists OR AB pharmacists OR TI pharmacist OR AB pharmacist	
4	(MH "Adult+") OR (MH "Expectant Mothers") OR (MH "Parents+") OR TI adult OR AB adult OR TI adults OR AB adults OR TI parenting OR AB parenting OR TI parent OR AB parent OR TI parents OR AB parents OR TI mother OR AB mother OR TI father OR AB father OR TI mothers OR AB mothers OR TI fathers OR AB fathers OR TI parenthood OR AB parenthood OR TI parental OR AB parental OR TI Patient OR AB Patient OR TI patients OR AB patients OR ((TI pregnant OR AB pregnant OR TI pregnancy OR AB pregnancy) AND (TI woman OR AB woman OR TI women OR AB women OR TI person OR AB person OR TI persons OR AB persons))	3,031,917
5	TI recommend OR AB recommend OR TI recommends OR AB recommends OR TI recommendation OR AB recommendation OR TI recommendations OR AB recommendations OR TI recommending OR AB recommending OR TI recommended OR AB recommended OR TI communication OR AB communication OR TI communications OR AB communications OR TI communicated OR AB communicated OR TI communicate OR AB communicate OR TI communicating OR AB communicating OR TI communicates OR AB communicates	340,660
	1 AND 2 AND 3 AND 4 AND 5	1,098

Database: APA PsycINFO via EBSCO

Set #		Results
1	DE "Immunization" OR TI vaccine OR AB vaccine OR TI vaccines OR AB vaccines OR TI vaccination OR AB vaccination OR TI vaccinate OR AB vaccinate OR TI vaccinated OR AB vaccinated OR TI immunization OR AB immunization OR TI immunizations OR AB immunizations OR TI immunisation OR AB immunisation OR TI immunisations OR AB immunisations OR TI immunise OR AB immunise OR TI immunises OR AB immunises OR TI immunised OR AB immunised	8,646
2	DE "Health Attitudes" OR DE "Public Health Attitudes" OR TI attitudes OR AB attitudes OR TI attitude OR AB attitude OR TI avoidance OR AB avoidance OR TI avoids OR AB avoids OR TI avoid OR AB avoid OR TI avoided OR AB avoided OR TI choices OR AB choices OR TI choice OR AB choice OR TI choose OR AB choose OR TI choosing OR AB choosing OR TI concern OR AB concern OR TI concerns OR AB concerns OR TI concerned OR AB concerned OR TI confidence OR AB confidence OR TI confident OR AB confident OR TI decision OR AB decision OR TI decisions OR AB decisions OR TI decide OR AB decide OR TI decided OR AB decided OR TI deciding OR AB deciding OR TI decides OR AB decides OR TI delay OR AB delay OR TI delays OR AB delays OR TI delaying OR AB delaying OR TI delayed OR AB delayed OR TI distrust OR AB distrust OR TI distrusting OR AB distrusting OR TI distrusted OR AB distrusted OR TI doubt OR AB doubt OR TI doubts OR AB doubts OR TI doubting OR AB doubting OR TI doubted OR AB doubted OR TI fear OR AB fear OR TI fears OR AB fears OR TI fearing OR AB fearing OR TI hesitation OR AB hesitation OR TI hesitates OR AB hesitates OR TI hesitate OR AB hesitate OR TI hesitated OR AB hesitated OR TI hesitancy OR AB hesitancy OR TI knowledge OR AB knowledge OR TI misconception OR AB misconception OR TI misinformation OR AB misinformation OR TI misinformed OR AB misinformed OR TI mistrust OR AB mistrust OR TI opposition OR AB opposition OR TI oppose OR AB oppose OR TI opposing OR AB opposing OR TI opposes OR AB opposes OR TI opposed OR AB opposed OR TI perceived OR AB perceived OR TI perception OR AB perception OR TI perceptions OR AB perceptions OR TI refusal OR AB refusal OR TI refuses OR AB refuses OR TI refused OR AB refused OR TI refusals OR AB refusals OR TI refusing OR AB refusing OR TI refuse OR AB refuse OR TI rejection OR AB rejection OR TI rejects OR AB rejects OR TI reject OR AB reject OR TI rejected OR AB rejected OR TI trust OR AB trust OR TI trusts OR AB trusts OR TI trusted OR AB trusted OR TI trusting OR AB trusting OR TI uptake OR AB uptake	1,583,304
3	DE "Health Personnel Attitudes" OR DE "Health Personnel" OR TI "healthcare worker" OR AB "healthcare worker" OR TI "healthcare workers" OR AB "healthcare workers" OR TI "healthcare provider" OR AB "healthcare provider" OR TI "healthcare providers" OR AB "healthcare providers" OR TI "healthcare professionals" OR AB "healthcare professionals" OR TI "healthcare professional" OR AB "healthcare professional" OR TI practitioner OR AB practitioner OR TI practitioners OR AB practitioners OR TI physician OR AB physician OR TI physicians OR AB physicians OR TI doctor OR AB doctor OR TI doctors OR AB doctors OR TI clinician OR AB clinician OR TI clinicians OR AB clinicians OR TI HCP OR AB HCP OR TI HCPs OR AB HCPs OR TI nurse OR AB nurse OR TI nurses OR AB nurses OR TI "physician assistant" OR AB "physician assistant" OR TI "physician's assistant" OR AB "physician's assistant" OR TI "physician assistants" OR AB "physician assistants" OR TI "physician's assistants" OR AB "physician's assistants" OR TI pharmacists OR AB pharmacists OR TI pharmacist OR AB pharmacist	328,833
4	DE "Parents" OR TI adult OR AB adult OR TI adults OR AB adults OR TI parenting OR AB parenting OR TI parent OR AB parent OR TI parents OR AB parents OR TI mother OR AB mother OR TI father OR AB father OR TI mothers OR AB mothers OR TI fathers OR AB fathers OR TI parenthood OR AB parenthood OR TI parental OR AB parental OR TI Patient OR AB Patient OR TI patients OR AB patients OR ((TI pregnant OR AB pregnant OR TI pregnancy OR AB pregnancy) AND (TI woman OR AB woman OR TI women OR AB women OR TI person OR AB person OR TI persons OR AB persons))	1,352,610
5	TI recommend OR AB recommend OR TI recommends OR AB recommends OR TI recommendation OR AB recommendation OR TI recommendations OR AB recommendations OR TI recommending OR AB recommending OR TI recommended OR AB recommended OR TI communication OR AB communication OR TI communications	351,736

	OR AB communications OR TI communicated OR AB communicated OR TI communicate OR AB communicate OR TI communicating OR AB communicating OR TI communicates OR AB communicates	
	1 AND 2 AND 3 AND 4 AND 5	327

Supplementary 2: Summary Table of Included Studies

Author*, publication year, country of study	Vaccine(s) studied	Research design	Sample size (response rate)	a) HCP groups or specialties b) Patient population	Factors found positively [+], negatively [-], or not [x] associated with HCP recommending vaccines or discussing diseases/vaccines with patients	Additional relevant findings
**Allison et al., 2013, US[76]**	HPV	Survey on physician attitudes, practice, and barriers concerning HPV4	609 (72%)	a) Pediatricians and FM b) 11- to 12-year-old boys	[+] physicians who routinely discuss sexual activity with 11-12-yr boys, higher perception of HPV severity; patients aged 13-15 and 16-18 vs. 11-12. [-] rural practice location, disagreed or not know HPV4 prevents genital warts in males. [x] no difference between pediatricians and FM	Majority of physicians thought HPV4 in boys was justified to protect both boys and their future female partners, but few reported recommending or administering HPV4 to boys
**Allison et al., 2016, US[83]**	HPV	Self-developed questionnaire (SDQ) w/21 Q on physicians' knowledge and attitudes, estimates of parents deferring vaccine and reasons	582 (70%)	a) FP & pediatrician b) 11-12 & 13-15 boys & girls	[+] older, girls. >25% parents defer vaccinate 11-12 child when offered. [-] physicians who rarely/never discuss more likely be FP (aOR 2.0) male (aOR: 1.8), disagree parents are more likely to accept if discussed w/other vaccines (aOR: 2.3), report 25%-49% (aOR: 2.8) or ≥50% (aOR: 7.8) of parents defer for child at 11-12 years, express concern of waning immunity (aOR: 3.4). [x] Practice location, setting, knowledge that HPV vaccine produces stronger antibodies in younger adolescents, believe necessary to discuss sexual health issues, specialty, physician gender	60% pediatricians (Ped) & 59% FP strongly recommend; 84% Ped & 75% FP frequently/always discuss at 11-12 year well visit. Reasons for not: expect or experienced that parents defer or not accept, patient too young or not yet sexually active, no time.
**Almughais et al., 2018, Saudi Arabia[97]**	HPV	E-mail survey on perceptions, measured knowledge w/a summary score of factual questions	200 (87%)	a) Full time PHC physicians	[+] being female (OR 3.1), knowledge, acknowledging vaccine as public safety issue (OR 4.8); [x] age, knowledge about infections	More female physicians believed in importance of HPV vaccine (84.7%), vs. male physicians (73.0%). Younger physicians view HPV vaccine as a necessary public safety issue (88.2%) compared to older physicians (73%), p=0.009. Almost 80% of study participants perceived HPV vaccine as necessary/important to public safety, while only 16.5% actually recommend to patients.
**Arnell et al., 2019, US[120]**	HPV	E-mail survey on knowledge, attitudes, and practices (KAP)	266 (28.6%)	a) Dentists and dental hygienists b) Dental patients	[+] being a dentist, knowledge scores and discussing (r = .213) and recommending (r=0.209)	Negative correlation between knowledge scores and believing HPV would encourage risky behavior (r = − .251), lack of confidence (r = − .296), and safety and efficacy concerns (r = − .277)
**Barnack et al., 2010, US[119]**	HPV	E-mail survey assessing	100	a) Pediatricians, PCP, and gynecologists	[+] being a pediatrician rather than a general practitioner, specialty other than pediatrician,	About 2/3 of respondents believed the cost of the vaccine is a barrier for parents to vaccinate their children.

		attitudes and practices		b) Parents of child ages 7–17	OB/GYN, or general practitioner, vaccinating own children	
**Bean and Catania, 2013, US[36]**	Vaccines in general	† Qualitative study using interviews to assess vaccination beliefs, perceptions and practice	15	a) Traditional providers (allopathic or osteopathic physicians/pediatricians, nurses) and alternative providers (chiropractors, midwives)<br>b) Adult patients with children, pregnant women, children	[+] Vaccine Supporters: view vaccine as effective, accept without reservation, believe benefits outweigh risks/vaccines protect against serious diseases/herd immunity prevents spread of disease, having experience with vaccine preventable disease, strongly influenced by norms; [-] Vaccine Opposers: believe disease-acquired immunity is preferable to vaccine-conferred immunity/vaccines could cause health risks, desire to maximize safety of patients, little personal experience with vaccine preventable disease, dangers of vaccines in combination	20% fully supported all recommended vaccines, 33% completely opposed vaccines, and 47% questioned or opposed 1 or more vaccines but supported others (vaccine conditional group). Some respondents in the vaccine conditional group questioned efficacy, dosing, and timing, but support was given by some for herd immunity and protecting against illness. 80% believed profit was a motive for vaccine development and promotion.
**Betsch and Wicker, 2014, Germany[126]**	MMR, influenza, hepatitis, pneumococcus	Questionnaire assessing KAP	135 (55.7%)	a) Occupational physicians<br>b) HCPs and pregnant women	[+] more positive attitudes towards vaccination (OR = 1.86), less misconceptions (OR = 1.39); [x] knowledge regarding official recommendations did not influence vaccine provision, board certification	Greater knowledge related to a more favorable attitude ($r = 0.245$, $p < 0.01$). Believed probability of adverse events to be low and of minor severity. Only vaccinations against hepatitis B and influenza are recommended unanimously to HCP (variance in recommendations for other vaccine types)
**Böhm et al., 2019, Germany[63]**	Influenza, pertussis	Paper or online questionnaire assessing attitudes, practices and barriers	867 (11%)	a) Gynecologists in private practice<br>b) Pregnant women	[+] informing patients about vaccines outside of patient request; [-] perceiving more barriers for pertussis than influenza vaccine and recommendation of pertussis vaccine, believing integrating vaccination into practice is a barrier, believing ASHIP restrictions are a barrier for recommending and providing vaccine, perceive safety and efficacy as barriers; [x] sex, age, years of experience, region	Physicians believing that a lack of STIKO recommendation, limited effectiveness, safety concerns and logistical difficulties were vaccination barriers were least likely to perform pertussis vaccination. Participants not vaccinating pregnant women against pertussis would if STIKO recommended it, but this was lower for participants who did not believe in the vaccine's efficacy and safety or believed infant pertussis is low risk.
**Bovier et al., 2005, Switzerland [94]**	Rubella, DTap, polio, Hepatitis B, influenza, Hepatitis A, Measles, Pneumococcal diseases	Mail survey on perceived utility, reported use, attitudes, patient or organizational barriers	1166 (64%)	a) Community based PCP	[-] reporting a barrier associated with lower vaccine use; [x] score of use not related to age, sex, practice characteristics, agreeing vaccination was a task of specialized centers, that the vaccine is promoted adequately at the national level, and that the patient says a categorical no to vaccination	Practitioners from a German-speaking region or without specialty qualification scored lower on utility of recommended vaccinations. Association between regular use of the different sources of recommendations and scoring higher on the vaccination utility score, except when colleagues served as the source of recommendation.
**Bruno et al., 2015, US[90]**	HPV	Mail survey assessing knowledge, attitudes, beliefs, barriers	121 (77.6%)	a) PCPs (pediatricians, FP, internists)<br>b) Minority populations in Brooklyn, N.Y.	[+] believing infection/complication serious enough for vaccine; [x] gender, age, language, country of origin, perceived patient barriers, perceived provider barriers, believing children receive too many vaccines/offering HPV	N/A

					vaccine opens door for sex education, being unsure about immunity	
**Bynum et al., 2014, US[66]**	HPV	Survey assessing practices and barriers	433 (68.3%)	a) Physicians b) Medicaid-enrolled adolescents in 3 groups: early (9-10), target (11-12), early adolescent catch-up (13-14), late adolescent catch-up (15-17)	[-] discomfort discussing STIs w/early (OR=1.75) and target adolescents (OR=2.75), difficulty ensuring vaccine completion for early adolescents (OR=0.73), discomfort discussing STIs w/parents of early (OR=0.44), target (OR=0.33), and catch-up adolescents (OR=0.47), time constraints (OR=0.70) and efficacy concerns (OR=0.65) for target adolescents, for early adolescents obstetrics/gynecology (OR=0.25), FM (OR=0.24), primarily non-Hispanic black patients (OR=0.15), concern about practice riskier behaviors (OR=0.57), FM (OR=0.20); [x] physician race/ethnicity, concerns about safety, adding another vaccine to schedule, lack of vaccine information, cost of stocking vaccine, reimbursement, vaccine being optional for school attendance	No factors were associated with recommendation of the HPV vaccine across all four age groups.
**Canon et al., 2017, India[131]**	HPV	Questionnaire assessing KAP	210 (70%)	a) Pediatrics, obstetrics/ gynecology, FM, IM, homeopathic medicine	[+] vaccine free from a government sector; [-] uncertainty about whether HPV must be persistent to cause cervical cancer or not; [x] age, gender, patient payment, CME course offered, belief it makes youth more promiscuous, belief it prevents vulvar or cervical cancer	Academic physicians would not recommend the vaccine due to specific concerns such as cost, side effects, and efficacy, but community physicians were not sure why they would not recommend the vaccine.
**Collange et al., 2016, France[54]**	HPV	Questionnaire assessing KAP	1712 (46%)	a) General practitioners b) Adolescent girls	[+] having higher workloads (OR=1.63-1.74), seeing patients w/cervical cancer in past 5 years (1.46); [-] unfavorable perception of risk-benefit balance (OR=0.94), doubts about vaccine utility (OR=0.78), not vaccinating own daughter (OR=0.13), not having a daughter aged 11-25 (OR=0.43); [x] trust in official sources about vaccination, perceptions of barriers to HPV vaccine, practice of alternative medicine, gender, years in practice	88.6% reported no difficulties in talking about sexual issues with young girls during consultations. 26.9% felt that a parent's presence was a problem, and 60.5% believed not enough is known about the vaccine's risks. 23.5% reported doubts about its efficacy. 26.4% considered some of the vaccines recommended by authorities today useless. 20.1% believe children are vaccinated against too many diseases.
**Daley et al., 2006, US[99]**	HPV	Cross-sectional survey assessing attitudes, knowledge, barriers (conducted before	294 (68%)	a) Pediatricians b) Young female adolescent (10-12 years old)	[+] knowledge that HPV vaccines are developing (AOR=2.33), belief that other recommendations facilitate introducing HPV vaccine (AOR=1.86), discussing sexuality before recommending (AOR=0.42), to older adolescents, female patients; [-] male physician, discomfort discussing sexuality w/female patients (p=0.05), not prescribing routine oral contraceptives in their practice,	Female physicians more likely than males to anticipate vaccinating young male adolescents (OR=2.03) but not female patients. 77% thought inadequate reimbursement would be a barrier, 51% thought that the "up-front" costs for the practice would be a barrier, and 57% and 64% thought that parental refusals would be a barrier for female patients and male patients, respectively.

		vaccine licensure)			concern about parent beliefs (AOR=0.29), parents' refusing vaccination as barrier (AOR=0.53); [x] attitudes about sexuality, comfort discussing sex, perception of sexual activity among patients, degree of knowledge, perceptions about parents	
**Davis et al., 2003, US[60]**	Pneumo-coccal conjugate	Separate surveys for physicians who adopted PCV7 recommendations and those who did not--assessing attitudes, practices, experiences	788 (60%)	a) FP, pediatricians b) Children less than or equal to 5 years of age	[+] larger practices, caring for more newborns, practices w/higher proportions of African-American children or Medicaid beneficiaries, female physician, consider giving ≥4 vaccine injections at 1 visit, expecting PCV7 to be effective, experienced patient death or morbidity caused by pneumococcal disease, spend more time discussing vaccine risks, PCV7 for children with recurrent otitis media/those in day care; [-] report parent concern about multiple injection, concern that multiple injections cause adverse effects/too much pain; [x] state financing strategies for PCV7, practice ownership, awareness of nurses' concerns	Almost all who administered PCV7 recommended the vaccine for immuno-compromised children.
**Dombkowski et al., 2008, US[117]**	Influenza	Survey assessing attitudes and experiences	389 (67%)	a) GP and FP in primary care offices	[+] being a pediatrician (AOR=3.49), having > 25 pediatric asthma patients (for patients with persistent (OR=20.6) and intermittent (OR=2.41) asthma), stocking privately purchased vaccine for patients w/persistent asthma (OR=6.1), stocking publicly purchased for patients w/intermittent asthma (OR=2.1); [-] believing asthma not severe enough to warrant vaccination, not discussing influenza vaccine at time of visit, child not identified as eligible for vaccine; [x] number of physicians in practice, practice affiliation, percentage of patients in Medicaid	Physicians who provide care for 25 or fewer pediatric asthma patients and those in smaller practices more likely to report child's asthma was not severe enough to warrant vaccination. Reported reasons why a child did not receive the vaccine were unavailability of vaccine at time of visit (49%), child had received vaccine elsewhere (46%), and child or parent refusal of vaccine (44%).
**Dubé et al., 2010, Canada[92]**	H1N1	Mail-based questionnaire assessing KAP	921 (31%)	a) Family physicians, pediatricians	[+] own intended uptake, believe the vaccine would be well accepted, belief that A(H1N1) generates significant health/ economic burden, belief that vaccine is safe/A(H1N1) is a serious disease/seasonal influenza vaccines are useful, being influenced by recommendations of professional associations of new vaccines	88% of family physicians and 90% of pediatricians intended to strongly recommend. 77% of family physicians and 83% of pediatricians intended to get vaccinated against A(H1N1) themselves. 47% of family physicians and 36% of pediatricians considered their vaccine knowledge to be insufficient.
**Dubé et al., 2011, Canada[74]**	Pneumo-coccal conjugate	Mail-based questionnaire developed using Health Belief Model	912 (50%)	a) Pediatricians b) Children under 3 years old	[+] vaccines administered each month in place of practice, perceived safety and efficacy of new PCV vaccines, perceived seriousness/frequency/economic burden of vaccine-preventable pneumococcal diseases,	Respondents who disagreed with PCV safety more frequently reported adverse events as a barrier, but 99% considered new PCV to be safe. Principle barrier to vaccination was perceived risks. 70% believe they have sufficient knowledge of the

		(HBM) and the Analytical Framework assessing knowledge, attitudes and beliefs			perceived acceptability of the vaccines, self-estimated sufficiency of knowledge on new pneumococcal vaccines; [-] disagreeing that the protection of other children against transmission of infection encourages recommendation practice; [x] perceived severity of Acute otitis media (AoM)/susceptibility to AOM, location, number of hours per week in outpatient	vaccine. 91% said their recommendations were influenced by expert groups, 90% by professional organizations, 88% by own knowledge.
**Dubé et al., 2011, Canada[40]**	Rotavirus	Mail-based questionnaire developed using Health Belief Model (HBM) and the Analytical framework assessing knowledge, attitudes and beliefs	912 (50%)	a) Pediatricians b) Children under 3 years old	[+] perceived health/economic burden of rotavirus, perceived acceptability/safety/efficacy of rotavirus vaccines, perceived severity of rotavirus infections, perceived benefits of rotavirus prevention by vaccination, self-estimated knowledge, perceived usefulness of a publicly funded program (r = 0.74), [-] cost of vaccine (r=−0.22), perception of low acceptability by parents	53% strongly intended to recommend rotavirus vaccines. Respondents that reported sufficient knowledge more likely to consider rotavirus vaccines safe and effective. 59% believe they have sufficient knowledge on the vaccines. 91% were highly influenced by recommendations made by expert groups, 90% by professional associations, and 88% by their personal knowledge. Reducing severe gastroenteritis was viewed as a benefit to rotavirus vaccination while risk of adverse events was a main barrier. <30% believed the vaccine is well accepted by parents.
**Dubé et al., 2011, Canada[39]**	A(H1N1)	Mail-based questionnaire developed using the Analytic Framework assessing KAP	912 (50%)	a) Pediatricians	[+] own intended uptake (OR=8.65), belief the vaccine would be well-accepted (OR=6.65) or is very useful to protect children (OR=2.84), belief A(H1N1) pandemic influenza causes economic burden (OR=2.78), knowledge of vaccine (OR=2.10), belief that the vaccine is safe (OR=2.10) and that A(H1N1) pandemic influenza is serious (OR=2.09) and severe (OR=1.88); [x] responding before or after vaccine campaign	Intention to recommend increased from 80% before the beginning of the campaign to 92% after. 40% of those who disagreed with the usefulness of the seasonal influenza vaccine did not intended to recommend, but only 2% of those who agreed did not intend to recommend it. 91% said their recommendations are influenced by expert group recommendations, and 90% said professional association recommendations. Increase in participants believing the vaccine is safe (50%-77%) and effective (35%-72%) after campaign. Decrease in respondents who were not sure if they would be vaccinated themselves after campaign (13%-3%).
**Dubé et al., 2019, Canada[55]**	Influenza	Web-based questionnaire assessing KAP	1,061	a) Maternity Care Providers (FP, obstetricians-gynecologists, midwives, pharmacists, nurses who care for pregnant individuals)	[+] followed official recommendations last season (AOR=3.6), discussed vaccination in general with most or all pregnant patients (AOR=3.2), have been vaccinated in previous influenza season (AOR=3.1), consider vaccine safe for fetus (AOR=2.7), self-evaluated knowledge (AOR=2.4), working in a practice where vaccines are administered (AOR=2.4), confident to offer advice (AOR=1.7), received information on vaccination during pregnancy during previous season (AOR=1.7)	Reasons for not recommending were low efficacy, safety concerns, and lack of information. Nurses and family-physicians had higher self-reported knowledge of vaccines in general than OB-GYNs, pharmacists, or midwives.

				b) Pregnant women		
**Duval et al., 2009, Canada[41]**	HPV	SAQ developed w/ Systems Model of Clinical Preventive Care on knowledge, attitudes and intentions	946 (53%)	a) Nurses working in community health centers, hospitals, private offices	[+] administering vaccines at workplace, knowledge (OR=1.9), patients' compliance (OR=4.9), sufficient information (OR=2.8), parents' acceptance of HPV vaccination for their children (OR=3.8), nurses' support of HPV vaccination (OR=3.0), working with children/teenagers (OR=2.2), providing information about vaccines at workplace (OR=3.1), believing the best age for universal immunization is <14 (OR=2.7)	Those with a lower-than-average knowledge score were more likely to think best age for a universal immunization would be 14–17 years. Higher proportion would support the vaccine if it was publicly funded. 82% believed all sexually active females should receive the vaccine.
**Ehresmann et al., 2000, US[84]**	Varicella	Survey assessing attitudes, practices, and beliefs	255 (78%)	a) FP and pediatricians b) Children aged 12-18 months	[+] pediatricians rather than family physicians (OR=2.0), believing recommendation of the AAP/AAFP is "very important" (OR=2.9); [-] preferring natural varicella disease over vaccine (OR=0.3), concern over waning immunity (OR=0.3); [x] attitudes toward varicella disease severity, number of childhood injections	Pediatricians more likely than FP to believe recommendations from professional organization (OR=2.6) and parental requests (OR=1.9) as "very important." Parent requests considered "very important" in recommendation for those who do not routinely offer the vaccine, but it was less frequently seen as "very important" for those who do offer it.
**Esposito et al., 2007, Italy[81]**	HPV	Anonymous SAQ assessing KAP	311 (77.8%)	a) Physicians b) Females aged 11–12 years	[+] being ≥45 years old (OR=4.64-5.34) or pediatric resident (OR=4.19), believing vaccine is useful for preventing cancer (OR=3.96, that the vaccine should be recommended before sexual activity (OR=2.27), discussing sexuality (OR=4.89), believing pediatricians have role in vaccine acceptance (OR=4.01); for 11–12-year-old patients [+] knowing two HPV vaccines were or would soon be registered (OR=2.04)	Primary care pediatricians had poorest knowledge, but pediatric residents had the most knowledge. 84.8% intended to recommend the vaccine and 60.6% of this group believed males and females should both be vaccinated. Majority did not discuss sexuality with patients or parents unless they were asked to or the patient had a clinical problem.
**Fagnan et al., 2011, US[116]**	Childhood vaccinations	Mail-based questionnaire to assess practices, beliefs, and barriers	413 (58%)	a) Physicians, NP, PA b) Children (0-36 months)	[+] pediatricians more likely to provide rather than refer out, adopting best practices, participating in Vaccine for Children program, having a system to track patient immunization status, having reviewed status of patient population; [-] rural area; [x] disciple, years practicing, screening immunization status at all visits, sending out reminders	About 1 in 5 referred out for all immunizations. Reasons: documentation hassles, inadequate staffing, language barriers, inconvenience to counsel patients, and concerns about vaccine safety. Reasons for not providing vaccine: too difficult to store (73%), absence of school requirements (46%), too new (20%). Referral was common for varicella (82%), influenza (6%), pneumococcus (86%) and less common for DTaP, IPV, MMR, Hib, and Hepatitis B.
**Farias et al., 2017, US[61]**	HPV	Online survey via email on KAP & electronic medical records of vaccination	134 (59.8%)	a) Pediatricians b) 11–18-year-old patients	[+] higher vaccine initiation for female physicians; [-] physician concern about financial burden for patient (RR=0.76), safety (RR=0.76), and vaccine efficacy (RR=0.73), older physicians	Barriers included discomfort talking about STIs with parents and patients, vaccine safety, HPV vaccine is not required for school attendance, difficulty ensuring completion of 3-dose vaccine series, and infrequent office visits by adolescent patients.

**Feemster et al., 2008[77]**	HPV	Online questionnaire assessing KAP	105 (59%)	a) Pediatricians<br>b) 11–12-year-old girls	[+] adopting new technology early (AOR=1.63), anticipating parental safety concerns (AOR=1.55); [-] concerns about safety (AOR=0.54); [x] anticipated parental concerns or provider concerns regarding sexuality	
**Ferrara et al., 2018, Italy[93]**	Meningococcal B	Online questionnaire assessing KAP	200 (28.6%)	a) Pediatricians<br>b) Children 2 years old or younger	[+] positive attitude toward utility of the 4CMenB vaccine (OR=5.28), confidence of knowledge about vaccine (OR=4.02), correct knowledge of schedule (OR=3.71); [x] practice setting	Younger participants (OR=0.78) and participants working a high number of hours per week (OR=1.06) had higher knowledge. A positive attitude toward vaccine utility was observed in participants that were younger (OR=0.81), graduated earlier (OR=1.29), worked in a primary care sector (OR=3.58), saw less patients weekly (OR=0.99), knew the vaccine was not included in Vaccine Action Plans (OR=2.14), and got information from guidelines and scientific journals (OR=2.88).
**Flicoteaux et al., 2014, France[89]**	Pandemic vaccination (pvaccination)	Survey conducted through a telephone interview assessing KAP for vaccinating all adults or at-risk adults only	1431 (36.8%)	a) GP<br>b) Adults ≤65 years of age	[+] consulting official information sources (to all adults: OR=2.03, to at-risk adults: OR=1.78), patient hospitalized from pandemic influenza (all adults: OR= 2.81);<br>[-] consulting news media for information (all adults: OR=0.57; at risk adults: OR=0.72), perceived low pandemic severity (all adults: OR=0.6), fear of side effects (all adults: OR=0.17; at-risk adults: OR=0.46), doubting vaccine efficacy (all adults: OR=0.28; at-risk adults: OR= 0.57), fear of side effects/doubting efficacy (all adults: OR=0.13; at-risk adults: OR=0.33);<br>[x] for at risk adults, perceived low severity of pandemic/patient hospitalization for influenza	33.3% had recommended the vaccine to at-risk adults only. 40.1% had recommended the vaccine to all adults. 26.6% did not recommend to any adult. 71.5% received the seasonal flu vaccine every year since 2007. Regarding attitudes toward the vaccine for themselves, 32.6% feared side effects, 45.3% doubted the vaccine's efficacy, 51.9% thought the severity of the pandemic was low.
**Gesser-Edelsburg et al., 2017, Israel[88]**	Tdap and influenza immune-zation	Questionnaire assessing KAP	150	a) FP, attending or resident physicians at obstetrics and gynecology departments<br>b) Pregnant women	[+] believing the Tdap vaccine is safe, believing the influenza vaccine is safe; [-] specialty	>92% knew about health ministry recommendation but >26% did not recommend to patients; 40% believe vaccines are dangerous but still recommended. Reasons for not recommending: safety and efficacy concerns, lack for information, and thought influenza not a dangerous disease and pregnant women were not more at risk than general population.
**Gilca et al., 2009, Canada[57]**	Childhood vaccinations	Mail-based SAQ to assess KAP developed and validated through interviews	299 (59.8%)	a) Nurses<br>b) Children	[+] being female provider, working in community health care; [x] perceived usefulness; for new PCV-10 vaccine specifically: [+] believing it is useful to vaccinate children (OR=4.04), nurses acceptance (OR=7.39), administering vaccines (OR=2.37), believing a vaccine program would	86-98% were willing to recommend a candidate vaccine. Majority believe in usefulness of vaccines recommended by public health authorities. 35-69% believed they had sufficient information to recommend a vaccine. >80% thought the vaccines would be accepted by health professionals and the

		and discussions with experts and nurses			be useful (OR=3.40), believing the vaccine is safe (OR=3.14);<br>[x] parents acceptance; For HA-HB, MMRV, DTaP-IPV-Hib-HBV, ACYW-135, HPV, and Rotaviral vaccines: [+] perceiving as useful, believing the vaccine has professional support, perceiving safety, vaccine efficacy, involvement in administration, practice setting, duration of experience in nursing, education degree	public. >70% believe the 5 vaccines are safe and protective.
**Glavier et al., 2019, France[82]**	Vaccines in general	10-Q questionnaire to assess barriers to vaccination and potential solutions	287 (17.8%)	a) GP<br>b) Cancer patients treated with chemotherapy	[+] willingness to receive education (OR=3.23); [x] years of medical practice, practice setting	92.8% pro-vaccine in general. Reasons for not vaccinating: inappropriate training (45.6%), lack of readily available information (35%), difficulties convincing patients (39.4%), patient refusal (23.9%), brevity of visits (14.4%), lack of vaccine efficacy during chemotherapy (6.7%). 19.2% thought patients were receiving vaccine prescriptions from their oncologist or hematologist. 73.9% desired more training on vaccination of chemotherapy patients.
**Gust et al., 2008, US[118]**	Vaccines in general	cross-sectional electronic SAQ to assess attitudes and behaviors	1251 (65%)	a) Pediatricians, family practitioners	[-] family practitioners, smaller practices, concerns about immunization, using the internet/magazine stories/pharmaceutical companies as trusted sources of information;<br>[x] confidence in discussing immunizations, personal uptake, gender, region, age, number of patients seen, trust in all other sources as trusted information sources	The three most trusted sources of health information were (1) medical journals, (2) other physicians, and (3) government health agencies.
**Hobeika et al., 2019, Lebanon[95]**	Multiple types of vaccines	40-Q questionnaire assessing practice and knowledge	114 (54.8%)	a) Obstetricians, gynecologists<br>b) Pregnant women and non-pregnant adolescents	[+] believing there is sufficient data to counsel pregnant women, own uptake	37.7% did not recommend vaccines to pregnant women; reasons included lack of clear guidelines (51.2%), lack of sufficient safety data (37.2%), logistical barriers (7.0%), believing the vaccine is useless (4.6%). 78.9% recommended vaccine to non-pregnant adolescents; reasons included absence of guidelines (80%), insufficient safety data (20%). Reasons for recommending HPV vaccine included protection from cervical cancer (98.9%), warts (71.3%), anal cancer (50.6%), and vulvar cancer (48.3%).
**Hofstetter et al., 2017[103]**	HPV	Web-based survey assessing knowledge, practices, and barriers	418 (18.8-53.9%)	a) Pediatric endocrinologists, hematologist/oncologists, pulmonologists, rheumatologists	[+] hematology/oncology (AOR=4.69) or rheumatology (AOR=6.55) subspecialty, seeing more adolescent patients with chronic conditions (AOR=1.01), discussing sexual health (AOR=2.53), checking patient vaccination status (AOR=3.83); [-] discomfort discussing sexual health (AOR=0.28), insufficient information (AOR=0.45), lacking	6.5% answered all knowledge questions correctly. 58.6% thought both primary and subspecialty care providers should discuss HPV vaccine with patients with chronic medical conditions, but of this group, only 60.9% sometimes or always did so.

					PCP-subspecialist communication (AOR=0.38); [x] gender, time spent in outpatient setting, practice type, location, region, proportion of adolescent patients, patient race/ethnicity/ insurance status, provider knowledge	
**Hopkins et al., 2009 England[124]**	HPV	40-Q online SAQ on attitudes and willingness to recommend	222 (23%)	a) GP, pediatrics, obstetricians & gynecologists<br>b) Girls ages 11-13	[+] comfort discussing sexuality with patients and parents, being in favor of vaccines, self-rated knowledge; [-] feeling vaccine encourages unsafe sex, low self-rated knowledge; [x] specialty, gender, parental status	Majority of responders (87%) were in favor of a national vaccination campaign, but few (38%) felt adequately informed about the vaccine and 55% demonstrated a lack of knowledge about the etiology of cervical cancer
**Hoque et al., 2016 South Africa[121]**	HPV	Anonymous SAQ assessing information and practices	320	a) doctors employed permanently at a local tertiary hospital	[+] mentioning HPV 6 and 11 are responsible for > 90% of anogenital warts (OR 5.677), having patients comply with HPV vaccination (OR 4.907), receiving sufficient information about HPV vaccination (OR 4.458);	Level of knowledge was low (average # of correct responses = 5.11 out of 11), but the HPV vaccine recommendation was very high; all but one of the respondents disapproved of giving the vaccine before becoming sexually active
**Hurley et al., 2008[85]**	Herpes zoster	Internet or mail SAQ about shingles prevention, HZ vaccine, and barriers	595 (69%)	a) PCPs<br>b) 60-79 years old	[+] strongly agreeing that HZ and PHN cause significant burden of disease in older patients (OR 2.75), patients 60-79 years of age, 80+ years of age, or severely debilitated patients 50-59 years of age; [-] insufficient information about protection (OR 0.40), storing vaccine in the freezer aa barrier (0.31 OR), thinking it was not needed by patients/that patients would be unwilling to pay out of pocket (OR 0.57); [x] concern about immunosuppressed household members of the patient, efficacy as barrier, patient concern about safety, purchasing cost, that vaccine will not be licensed for immunosuppressed patients	Physicians perceived a high level of burden from HZ and PHN and generally favored the HZ vaccine
**Inoue et al., 2011 Japan[112]**	Influenza A/H1N1	Anonymous mail survey about attitudes, uptake, and practices	515 (51.50%)	a) PCPs	for intention to receive: [+] number of patients seen per day, reporting receiving seasonal vaccination annually; for intention to recommend: [+] number of patients seen, number of patient contacts with H1N1, intention to receive vaccine; [x] gender, age, department or specialty, receiving seasonal influenza vaccination annually	Those with knowledge of the past US Guillain-Barre syndrome cases associated with influenza vaccination were less likely to recommend the novel vaccine to patients (OR 0.66, p = 0.031)
**Jaoude et al., 2018, Lebanon[101]**	HPV	Survey with 64 clinical vignettes	228 (28.8%)	a) OBGYN, Pediatrics, FM, Infectious Diseases<br>b) age 11-12, 13-17, or ≥18	[+] Physicians w/higher knowledge (AOR 3.4). OBGYN vs. pediatricians (AOR 0.5). Patients: female (AOR 6.8), from non-conservative background (AOR 2.1), sexually active. [x] patient's age, physician's age and gender	Majority physicians had insufficient knowledge: not know condoms not enough to prevent HPV (51.2%), HPV causing head and neck cancers (57.1%).

**Jaramillo et al., 2009, Mexico[73]**	H1N1	Paper-based SAQ to determine the acceptance of the new H1N1 vaccine	1097 (65.1%)	a) General and specialist physicians, medical residents, nurses, NP	[+] doctors vs. nurses, older age, years of experience, believing in susceptibility to and severity of disease (OR=2.57), believing in vaccine's efficacy (OR=9.07) and safety (OR=10.30), considering themselves at high risk for getting (OR=2.32) or transmitting (OR=1.99) the virus, own intended uptake (OR=2.03), trusting info from international organizations, ID specialist, and NVIC	Factors associated with own acceptance of vaccine included: [+] having colleagues who got the infection (OR=1.79), perceiving high infection risk, believing H1N1 is a serious disease (OR=1.69), believing vaccine is safe (OR=6.51) and effective (OR=5.65), and having received the seasonal influenza vaccine in the past 5 years (OR=3.43).
**Kahn et al., 2005 US[49]**	HPV	Mail survey assessing beliefs, attitudes, and intention to recommend	513 (60%)	a) Pediatricians, FP<br>b) Adolescents	[+] number of 10–15-year-old patients seen per week (OR 1.3), knowledge about HPV (1.079), percentage of 15-year-old patients who are sexually active (0.084), likelihood of following organizational recommendations of (0.834), older patients; [-] perceived barriers to recommending immunization (-0.203).	
**Kao et al., 2012 Taiwan[122]**	Tdap	55 multiple choice questions on knowledge, beliefs, and attitude outcomes	510 (25.20%)	a) Obstetricians and gynecologists<br>b) Postpartum women	[+] higher knowledge scores of disease, vaccination, rate change in disease incidence among adults as increased (OR 2.1), rating disease among newborns as highly severe (OR 2.9) or highly contagious (OR 3.3), and understanding current recommendations (OR 5.7); [x] rating the severity of pertussis among adolescents and adults, age group the respondent considered the main source of pertussis infection for infants	The three most frequent reasons for recommendation were (1) believing that vaccine benefits outweigh risks (68.6%), (2) trusting guidelines (57.2%); and (3) believing that the immunization program is convenient (57.9%).
**Karlsson et al., 2019 Finland[58]**	Vaccines in general	E-survey on beliefs about benefits and safety, general trust of HCPs, and own behavior	2962 (49%)	a) Doctors, nurses<br>b) Children	[+] confidents in benefits and safety (for recommending and vaccinating own children); [-] perceiving vaccines as less beneficial and safe (for vaccinating own children); whether they had received the vaccines themselves (for recommending to patients); [x] trust	Majority of HCWs had high confidence in vaccinations, but many reported low vaccination confidence.
**Kassianos et al., 2018 Bulgaria[51]**	Influenza	Online/paper survey with MoVac-flu and MovAd (for influenza vaccination acceptance & advocacy)	2476	a) General practitioners, specialists, nurses	[+] characterized by engaged sentiment (OR 39.6 for uptake); confident sentiment (for recommending); [x] profession, gender	Countries differed in how often they recommended the flu vaccination
**Kempe et al., 2007 US[42]**	Rotavirus	Mail or internet survey assessing knowledge,	305 (71%)	a) Pediatricians<br>b) Children	[+] if ACIP made a recommendation for routine rather than permissive vaccination, perception that rotavirus is common and potentially severe, confidence in prelicensure studies; [-] concerns about safety, general	

		beliefs, and attitudes			parent concerns; [x] practice setting, gender, age, difficulty obtaining supplies, lack of reimbursement	
**Kempe et al., 2018 US[104]**	Meningococcal B	Online survey assessing frequency of discussion and a list of possible factors	660 (72%)	a) Pediatricians, FP<br>b) Adolescents, young adults	[+] awareness of disease outbreaks, private practice, college-aged patients; [-] being only somewhat or not at all aware of the vaccine, practicing in a health maintenance organization; [x] gender, location, region, age, awareness of patients who have had meningococcal disease, proportion of black, Hispanic, Medicaid patients	Existence of a recommendation for another meningococcal vaccine (MenACWY) and the consistency of reimbursement were related to a lower likelihood of recommendation
**Klett-Tammen et al., 2016 Germany[53]**	Tdap, influenza, pneumococcal	Mail survey assessing KAP	1337 (13.40%)	a) GP, PA<br>b) 60 years and older	[+] not trusting the STIKO-recommendations, negative perceived benefit harm ratio of respective vaccine, working in West Germany (only applies to any, IV, and PV, but not TV)	More GP than PA felt sufficiently informed regarding adult vaccinations (90 % of GP vs. 79 % PA), stated to know guidelines (97% GP vs. 86 % PA), and to use them as a source of information (85% GP vs. 69% PA).
**Krishnaswamy et al., 2019 Australia[114]**	dTpa, influenza	Online survey developed w/input from participant population	870 (25%)	a) GP, midwives, obstetricians<br>b) Pregnant women	[+] personal history of vaccination, confidence in their vaccine knowledge, awareness of guidelines and belief in the safety of dTpa during pregnancy; [x] profession, years providing care (unless higher than 15), type of practice	70% of obstetricians working primarily in private practice believed discussing vaccinations to be their primary responsibility compared to 36% in public practice
**Lehmann et al., 2017, Netherlands [75]**	Pneumococcal disease, herpes zoster, and pertussis	Online survey based on qualitative interviews about beliefs and patient population	723 (6%)	a) GP<br>b) 60 years and older	[+] patients' comorbidities; positive attitude towards vaccination and perceived severity (correlations $p < 0.001$)	Predictive factors for attitude: general attitude towards vaccination as a preventive tool, offering people aged 80+ vaccination, vaccination during outbreak; perceived severity of herpes zoster and pneumonia, and prevalence of herpes zoster.
**Le Maréchal et al., 2017 France[79]**	Meningococcal C	1st telephone survey in a series of 5 national cross-sectional surveys on GP attitudes, perceptions, and practice	1712 (46%)	a) GP<br>b) infants 12 month and children/young adults 2-25	[+] own children vaccinated, believing official recommendations were clear/that vaccines in general are useful and safe, being favorable to vaccination in general, no doubts about efficacy; [x] gender, density of municipality of practice, workload, practice type, alternative medicine, perceiving responsibility to recommend, intermediate view of vaccine safety, having had a patient with meningitis	Two specific factors were found to be associated with catchup vaccination in multivariate analysis, and both involved the GPs' perception of how patients see the vaccine's benefits.
**Le Maréchal et al., 2018 France[105]**	Diphtheria, tetanus, pertussis, poliomyelitis, Haemophilus	Telephone survey on beliefs, attitudes, practices for self and family, and	1582 (92% (from a pre-existing panel))	a) GP<br>b) Children	[+] feeling comfortable talking to patients about the safety of vaccines, consulting the French vaccination guide; [x] gender, age, location, workload, type of practice, alternative medicine	Very high prevalence (94%) of postponed hexavalent vaccination due to a febrile minor illness

	influenzae type b, and hepatitis B	recommendations				
**Lutringer-Magnin et al., 2011 France[37]**	HPV	‡ Mixed methods (survey & interview) on KAP for various vaccines	279 for survey, from which 35 selected for interview	a) GP<br>b) Adolescent and young adult females	[+] having more than 50 consultations with women per week, age under 55 years, routinely recommending hepatitis B vaccination; [x] occupational area, department, practice area, gender, prevention advised, reported difficulties	
**MacDougall et al., 2015 Canada[38]**	Tdap	‡ Mixed methods (interview, survey, and focus group) modified from previous validated surveys covering KAP	1,167 for survey + 8 focus groups and 4 interviews	a) FP, pharmacists<br>b) Adults	[+] strongly agreeing that pertussis is a serious threat, confusion, attitude that pertussis is rare/vaccine is no longer needed, supporting pharmacists administering vaccines; [-] barrier in time to provide, not enough information from public officials, NACI guidelines not promoted adequately, feeling neutral on the rarity of pertussis, efficacy of Tdap, wanting to protect patients, feeling neutral on agreeing with guidelines, strongly disagreeing with the importance of the vaccine, trusting the recommendations, was negatively associated with offering the vaccine	Neither agreeing nor disagreeing with the attitudinal statements was most correlated with not routinely offering Tdap to patients.
**Massin et al., 2015, France[43]**	Influenza	computer-assisted telephone interviews on risk attitudes, professional characteristics, attitudes, practices	1,136 (36.8%)	a) GP<br>b) Young adults	[+] risk-averse GPs more often vaccinated against seasonal and pandemic influenza, recommended the pandemic influenza vaccination more often than more risk-tolerant colleagues; [-] risk tolerant, to patients without risk factors; [x] risk attitudes did not influence pandemic influenza vaccination recommendations to at-risk patients	A high volume of activity and a group practice were each associated with an increased probability of vaccination against seasonal influenza, whereas practicing alternative medicine decreased this probability.
**Mui et al., 2013, China (Hong Kong) [67]**	Pneumococcal conjugate	Mail survey on practices, patients, setting, and barriers against advising vaccination	937 (24.7%)	a) Hong Kong GPs<br>b) Patients aged 50-65+	[+] own intention to receive the vaccine; [-] number of pneumonia patients in last year; concerns about promoting the vaccine/marketing, it being unrelated to consultation & having limited time; safety concerns & illness severity perceptions were barriers for patients 65+; [x] influenza vaccination, perceived severity and safety concerns for patients > 65, # of barriers, affordability, practice setting, specialty	Participation in the EVSS is associated with recommendation to elderly (ORm = 2.296, p,0.001) but not the middle-aged (p = 0.05).
**Napolitano et al., 2018, Italy[72]**	HPV	46-Q online survey in Italian and English	234 (36.5%)	a) PCPs<br>b) 11–18-year-old boys and girls	[+] longer practice activity (OR = 1.12), working in solo practice (OR = 0.29), belief that the vaccine was effective for boys (OR = 1.61); [x] gender, age, lower number of female	Major reasons for recommending vaccine to 11–12-year-old boys: preventing HPV-related diseases (73%), effectiveness (51.4%), and vaccine safety (51.3%). PCPs who did not recommend cited:

		covering attitudes, practices, and information sources			patients aged 11-12, reporting need for additional information	vaccine for this group not actively recommended (88.7%), lack of time to talk with their patients and parents about infection/vaccination (10.6%) and concerns about efficacy or side effects (5.6%).
**Neufeind et al., 2020, Germany[106]**	Influenza, pertussis, hepatitis B	Computer-assisted telephone interviews developed based on pre-existing validated survey questions	700 (20.4%)	a) FP b) Adolescents, adults	[+] being vaccinated themselves, confidence, collective responsibility, constraints, and complacency associated with uptake, but only higher confidence was associated with recommendation; [-] being a member of the homeopathic physician society, older (in western Germany); [x] city size, age, gender, region, scientific society, main society, constraints/complacency/calculation/observations with recommendation	Most frequently reported barriers for influenza were poor vaccine efficacy (40.6%) and a poor risk-utility profile (35.9%). For measles, main reasons were regularly forgetting about it (41.8%), organizational constraints (25.4%), and the belief that vaccinating only upon patient request is sufficient (24.7%).
**Newman and Taylor, 1998, US[87]**	Varicella	3-part survey on whether participants followed recommend-dations, presenting dis/advantage of vaccine, and practice	345 (76%)	a) Washington state pediatricians b) Children	[+] stocking the vaccine, having cared for varicella patient, citing AAP as primary source of information, having seen a death from varicella, agreement with reducing disease complications/that parents will lose less time at work, disagreement with it being not needed/not cost effective [-] working in community health centers; [x] having experience with pneumonia, believing parents pay out of pocket, finding it costly to store, not knowing who to immunize, and believing fewer natural infections lead to a larger pool of susceptible adults	
**Nikolic et al., 2015, Serbia[78]**	HPV	20-minute, 28-Q survey in a room where pediatricians could not consult the literature or each other	137 (78.7%)	a) Pediatricians b) Adolescents	[-] reporting insufficient time, believing parents lacked information, and doubting efficacy; [x] knowledge, demographics, parental attitudes, feeling uncomfortable discussing the vaccine, thinking the recommendations change too often, the vaccine being expensive, not feeling sufficiently informed, believing boys should be involved, believing it encourages risky sex behavior and reduces condom use	Pediatricians who believe that they know enough about the HPV vaccine are 3-times more willing to recommend compared to those who do not believe in the efficacy of the vaccine.
**Noh et al., 2016, Korea[132]**	Influenza	Mail/online survey on experience and practice of influenza vaccination to pregnant women.	473 (38.4%)	a) Obstetrician-gynecologists b) Pregnant women	[+] information about increased hospitalization in pregnant women with influenza, knowledge of effect of maternal influenza vaccination on the prevention of febrile episodes; [-] information about the safety of maternal influenza vaccination to pregnant women and the perinatal outcomes	Major reason for avoiding recommendation was concern over the safety of influenza vaccination during pregnancy (85.0%).

**Pavia et al., 2003, Italy[125]**	Influenza and pneumo-coccal	Anonymous mail survey on KAP	148 (30.3%)	a) GP b) Elderly patients	[+] Knowledge that the vaccines are recommended for elderly associated with administration; fewer years in practice, relied on scientific journals as info sources, and more hours worked/week associated with positive attitudes; [x] info source and # of hours worked with knowledge; age and knowledge with attitudes; all variables except knowledge with administration	95.2% administered/recommended influenza vaccine, 46.9% use pneumococcal vaccine. Concern about side effects was the most common reason for not administering and recommending these vaccines. Age and number of years in practice positively associated with knowledge that the vaccines are recommended for the elderly.
**Pelullo et al., 2020, Italy[96]**	Hepatitis B, MMR, varicella, pertussis, and influenza	Anonymous, structured SAQ developed by the research team	412 (57.6%)	a) Healthcare professionals b) Patients, colleagues	[+] HCWs having a child, working in pediatric/neonatal wards, knowledge, reporting no need for further information about vaccinations (associated with believing that information received about vaccinations reliable), considering vaccinations reliable (recommending); [x] being favorable to vaccinations, considering vaccinations useful, information sources	58% of HCWs reported the need for additional information about vaccinations.
**Perkins and Clark, 2012, US[35]**	HPV	† Semi-structured Interview on perceptions, behaviors, and beliefs	34	a) PCP and NP b) Low-income minority patients	[+] describing more enabling than impeding factors; [x] self-reported volume of vaccine-eligible patients, years in practice, training, type of practice, gender	Providers who believed vaccines overall are safe (HPV in particular) reported that this motivated their recommendations for patients and their own families.
**Power et al., 2009, US[98]**	HPV, influenza	Mail survey on attitudes about vaccines and pregnancy, and barriers	394 (51.2%)	a) Obstetrician-gynecologists b) Pregnant women	[+] providing primary care, being female and agreeing that screening for vaccine-preventable diseases should be a part of their routine practice; [-] inadequate information on effects of vaccines on fetus	About one in three responding physicians agreed that we still do not know enough about the effects of vaccines on the fetus to administer them safely in pregnancy.
**Praphasiri et al., 2017, Thailand[68]**	Influenza	Mail survey with dichotomous and open-ended responses	580 (57%)	a) Physicians b) Pregnant women	[+] believing pregnant women are at increased risk, safety of vaccine, efficacy of vaccine, and awareness of MOPH guidelines; [x] priority of influenza, type of hospital, specialty, and sex	Physicians cited lack of awareness of MOPH recommendations for pregnant women (70%), refusal of vaccine by patients (63%), lack of vaccines in their facility (60%) and cost of vaccines not being covered by insurance (60%) as important barriers to vaccination of pregnant women.
**Raude et al., 2016, France[62]**	MMR, MenC, Hepatitis B, influenza, HPV	Mail survey based on Theoretical Domains Framework & interviews with GPs	1,582 (46%)	a) GP b) Children and adults	[+] trust in institutions (trust [-] associated with concerns about vaccine safety); [-] vaccine safety associated with recommendations; [x] gender, self-efficacy	Concerns about safety acted as a mediator between institutional trust and recommendation.
**Riedesel et al., 2005, US[50]**	HPV	Mail survey based on a	145 (15.5%)	a) Pediatricians b) 11, 14, 17-year-old patients	[+] being female, having stronger normative beliefs, higher HPV knowledge, and fewer perceived barriers associated with intention to	Female providers with experience treating adolescents intended to recommend vaccination against cervical cancer more often.

		validated tool on AKP			administer; [+] recommendation by the AAFP associated with intention against both types; [-] reluctance to talk with patients about STIs and sexuality associated with intention to recommend	
**Rutten et al., 2017, US[123]**	HPV	E-mail, modified HPV Immunization National Trends Survey; medical data for initiation & completion (1 or 3 doses)	280 (41%)	a) Physicians, GP's, and nurse practitioners b) children aged 9–18	[+] increased knowledge scores (AIRR = 1.05 for initiation, AIRR = 1.28 for completion); greater clinician barriers; [-] increasing perceived parental barriers (AIRR = 0.94 for initiation, AIRR = 0.90 for completion); [x] clinician barriers after adjusting for site-level patient characteristics	Clinician barriers became non-significant after adjusting for site-level patient characteristics
**Shibli et al., 2017, Israel[113]**	Childhood vaccinations	Anonymous SAQ distributed to HCPs at their workplace and collected later by researchers	218 (60%)	a) Healthcare professionals b) Children	[+] knowledge level associated with attitudes; [+] knowledge score, attitude score, age, and professional experience associated with patient recommendation; latter associations disappeared in multivariate analysis. [+] knowledge level, age, and workplace associated with the vaccination of own children; [x] attitudes and participation in advanced public health course, having children, religion, profession, nationality, professional experience, experience treating children	The mean knowledge scores of nurses who completed a public health course were higher than nurses who did not participate.
**Shibli et al., 2019, Israel[107]**	Childhood vaccinations	Anonymous SAQ distributed to HCPs at their workplace and collected later by researchers	271 (72.2%)	a) Pediatricians b) Children	[+] knowledge level, attitude score, the type of employment, and number of parents encountered in bivariate analysis, but only workplace and the number of parents encountered were correlated with recommendation when controlling for other variables; [x] difference between nurses and physicians, percentage of children vaccinated, religious/secular, and gender	Only 37.3% of participants answered all knowledge questions correctly.
**Soon et al., 2015, US[127]**	HPV	E-mail questionnaire adapted from a validated scale on attitudes, behaviors, and practice	73 (19%)	a) Hawai'i pediatricians and family physicians b) Children aged 11-12 years	[+] discussing issues of sexuality before recommendation; [-] perceiving the need to talk about sex as a barrier (OR 0.18); [-] viewing parent concern as a barrier; [x] physician characteristics/demographics, confidence, costs, parental barriers, the time it takes to discuss	Discussing issues of sexuality was a negative factor for male patients.

**Stefanoff et al., 2020, Poland[108]**	MMR, 7 recommended vaccines	Structured, anonymous computer-assisted phone survey	500 (19%)	a) PCPs b) Children	[+] using only scientific sources for information on vaccines and addressing vaccination myths; [x] self-assessed knowledge; sex, age group, province (except for Zachodniopomorskie), attending a training and using nonscientific sources	Least supportive PCPs had lower median age (51 vs. 56 years), were more frequently men (29% vs. 24%), and attended a workshop or conference on vaccinations less commonly in the previous year (50% vs. 64%, p = 0.064).
**Suryadevara et al., 2015, US[69]**	DTaP, MMR, IV, HPV	One-page SAQ on vaccine attitudes and practices	680 (75%)	a) Pediatricians b) Children	[-] concerns about influenza vaccine efficacy; providers who believed that HPV vaccine increases likelihood of unprotected sex; [x] influenza vaccine safety concerns, Tdap vaccine safety or efficacy concerns and recommendations of parental receipt of these vaccines	Thirty-five (5%) of the 680 healthcare providers attending AAP sponsored conferences state that they either do not recommend all of the standard pediatric vaccines or they do not routinely recommend influenza and/or HPV vaccine; only 2 (6%) of these 35 providers considered themselves to be “vaccine hesitant.”
**Taylor et al., 2014, Australia[70]**	Meningococcal B	15 Q on experience & AKP (modeled after a US study on rotavirus vaccine)	523 (30%)	a) Registered and practicing FPs in metropolitan and rural South Australia	[+] being full time workers, having past experience with IMD, and reporting the extra injection as a barrier were associated with discussing non-funded immunizations; [x] location of practice, teaching or training, gender, pediatric medicine, who gave the immunization, cost as a barrier, SES as a barrier, effectiveness/safety as barriers, and being a new vaccine as a barrier	Attitudes toward specific vaccines as well as immunization in general were strongly influenced by professional experience
**Tong et al., 2008, Canada[111]**	Influenza	Anonymous 51-item questionnaire on AKP with an attitude summary score	227 (34%)	a) Maternal care providers b) Pregnant women	[+] high levels of provider knowledge about influenza and maternal vaccination, positive attitudes towards influenza vaccination, increased age, being a family physician, and having been vaccinated against influenza	The most important factor in women accepting vaccine during pregnancy was having a recommendation from a provider, yet one of the barriers identified by providers was uncertainty about who is responsible for discussing, recommending, and administering the vaccine.
**Topuridze et al., 2010, Georgia[129]**	Hepatitis B	SAQ about awareness, risk factors for exposure, and attitudes	297 (95%)	a) Physicians, nurses b) Other HCWs	[+] having colleagues who had been vaccinated and the perceived safety of the vaccine; [-] believing that HCWs were not at greater risk of HBV infection than the general population	The strongest predictor of vaccine acceptance and willingness to recommend vaccination was concern about HBV vaccine safety and adverse events.
**Torun and Torun, 2010, Turkey[56]**	Influenza	Anonymous SAQ on paper from one hospital	718 (76.3%)	a) Healthcare workers from Istanbul governmental hospital	[+] being a doctor, seasonal vaccination history, own uptake, agreement w/ safety/efficacy of pandemic influenza vaccine, adequate self-reported knowledge, trust in the MoH, source of knowledge other than only media for recommendation; [+] trust in MoH (for own vaccination); [x] purchasing Tamiflu, age, existence of chronic illness, daily patient contact, having children at home, years in healthcare; using MoH and being a doctor not significantly	More vaccinated compared to non-vaccinated HCWs agreed that ‘Vaccine is effective in preventing pandemic influenza’, ‘vaccine is safe’ and ‘healthcare workers have a professional responsibility to get influenza A/H1N1 vaccine’ ($p < 0.001$).

					associated with recommending in logistic regression analysis	
**Tuckerman et al., 2020, Australia[71]**	Influenza	31-Q e/mail survey on uptake, experience, knowledge, attitudes, barriers, and future educational resources	298 (24.8%)	a) Pediatric specialists and GP<br>b) Children with special risk medical conditions (SRMC)	[+] own annual uptake (aOR 3.96), [+] confidence in understanding 'medically at risk' conditions (aOR 1.82) and perceived responsibility to provide recommendation (aOR 7.35); [-] being regional practicing (aOR 0.25); [x] perceived effectiveness/safety of vaccination, prioritization of vaccination, time practicing medicine, HCP type	Although GPs and pediatric specialists perceive influenza to be a serious infection in children with SRMC, a considerable sub-group do not recommend the influenza vaccine regularly to these children.
**Vadaparampil et al., 2013, US[109]**	HPV	27-Q survey adapted from a previous nat'l study w/ composite HPV knowledge score	433 (68.3%)	a) Florida FP and pediatricians<br>b) Female patients aged 9-17	[+] being a pediatrician, having a private practice, practicing in a single specialty, being VFC provider, using 2 or more strategies to ensure the series was completed, not referring out for HPV vaccination, having mainly non-Hispanic white patients; [x] age, sex, race, number of physicians, HPV knowledge, barriers	The prevalence of HPV vaccination ranged from 0% to 61.9% with disparities in low-income females, even in the absence of vaccine cost.
**Vadaparampil et al., 2016, US[52]**	HPV	49-item survey using previous items to assess recommendation	367 (51%)	a) Florida FP and pediatricians<br>b) Male patients aged 11-18	[+] pediatric specialty (aOR 2.55), being a VFC provider (aOR 5.43); ; [-] fewer (or lower) physician-reported barriers (aOR 0.94); physician concern about vaccine safety, concern about adding another vaccine to the schedule and trouble remembering to discuss the vaccine; recommendations lower for younger boys; [x] rarely seeing adolescent males, not completing the series, not required by schools, VFC program not covering teenage males, discussing sexual behavior	Physicians who participated in the VFC program were more likely to recommend vaccination than those not participating
**Verger et al., 2015, France[64]**	MMR, meningococcal meningitis C, HPV, hepatitis B, influenza	Interview developed standardized questionnaire about six specific vaccine situations	1,712 (46%)	a) GP<br>b) Children, adolescents, infants, adults with diabetes under 65 years	[+] being comfortable explaining benefits and risks to patients (OR = 1.87), trusting official sources of information highly (OR = 1.40); [-] believing serious adverse effects to be likely (OR = 0.71), doubting vaccine's utility (OR = 0.21); [x] trusting official sources about vaccination and opinions about potential severe adverse effects	GPs' recommendations to patients were significantly associated with their own vaccination behavior (2013–2014 seasonal influenza: ORa = 2.95; 3 doses or more of hepatitis B vaccine: 1.90)
**Verger et al., 2016, France[110]**	Influenza, dTPolio, hepatitis B, HPV, MMR, meningitis C	Interview developed standardized questionnaire about six specific	1,582 (46%)	a) GP<br>b) Children, adolescents, infants, adults with diabetes under 65 years	[-] practicing alternative medicine occasionally, those with no patients who had had one of the five included VPDs, those who had had patients with a serious health problem leading to hospitalization that might have been related to vaccination as well as those who did not adhere to seasonal influenza or dTPolio vaccine	The third cluster (high hesitancy or opposition) included 3% of GPs, most of whom considered links between vaccines and severe adverse effects likely or very likely, had doubts about vaccine usefulness, and recommended vaccines much less often than the average.

		vaccine situations			recommendations for themselves; [x] sex, density of practice	
**Vezzosi et al., 2019, Italy[44]**	Influenza, pneumococcus, herpes zoster	Anonymous online survey designed from similar studies	73 (26.6%)	a) GP<br>b) Adults aged 65 and older	[+] having previously received flu vaccine (mOR 5.44), proactive attitude for VZ (mOR 13.67); familiarity with herpes zoster (mOR 6.61) and recommending PNV (mOR 19.36); [-] self-reported knowledge gap (mOR 0.07)	Most frequently reported reason for not recommending was the lack of information (5/11, 45.5% for PNV and 19/25, 76.0% for VZ), followed by doubts on the vaccine efficacy (27.3% and 12.0% for PNV and VZ, respectively).
**Vilca et al., 2018, Spain[128]**	Influenza & pertussis during pregnancy	14-item SDQ on KAP: # antenatal visits/week, own uptake, recommendation, & beliefs	194 (23%)	a) Obstetricians and midwives<br>b) Pregnant women	[+] only MCP's own vaccination associated with prescribing influenza vaccine during second/third trimester (OR 3.70), knowledge associated with recommending influenza vaccine & practice; [x] professional category, private practice, # of antenatal visits/week	Main barriers reported: 25.9% concern of adverse events (30.8% midwives vs. 10% OB/GYN, lack of experience administering, disease no longer important, workload
**Wagner et al., 2015, Austria[130]**	Meningococcal B	SDQ with open ended text boxes and multiple choice	231 (14.20%)	a) Pediatricians<br>b) Children	[x] location, practice, resident vs. hospital based	Of those who would not implement the vaccine, 45.8% said this was due to the newness of the vaccine and their lack of personal experience with its use and 42.5% cited the absence of an explicit recommendation in the Austrian National Immunization Plan.
**Wilcox et al., 2019, UK[86]**	Respiratory syncytial virus	Survey of pregnant women and maternity HCPs on awareness & experience of RSV	513	a) Obstetricians and midwives<br>b) Pregnant women	[+] being obstetricians (OR: 2.50), study site, good understanding of RSV (OR: 4.42), perceiving RSV as extremely (OR: 4.85) or moderately/somewhat serious (OR: 4.16) associated with supporting RSV trials; [x] perceived frequency of RSV, perceived bronchiolitis severity/frequency, bronchiolitis familiarity, RSV experience, ethnicity, and having own children	More HCPs would support administration of the vaccine if it was routinely recommended: 47% definitely, 34% likely, 14% not sure, 4% unlikely and 0.5% very unlikely
**Wilcox et al., 2020, UK[115]**	Influenza & pertussis during pregnancy	Anonymous SDQ with open and closed response questions	1,586	a) GP<br>b) Pregnant women	[+] length of practice, reviewing pregnant women routinely as part of antenatal care, believe discussing vaccine is their responsibility, confident in knowledge of pertussis vaccination (OR: 3.52) and influenza vaccination (OR: 2.34); [x] specialization	45% suggested that midwives and/or Secondary Care should take greater responsibility in antenatal vaccination.
**Wong et al., 2017, Malaysia[100]**	HPV	23-item mail survey on background, experiences, and attitudes of HPV vaccine for boys	357 (22.5%)	a) Physicians providing HPV vaccine<br>b) Adolescent boys ages 11-18 years old	[+] practicing for 21 to 30 years and working in urban areas, numbers of years in practice and locality of clinic; [x] age group, gender, ethnicity of physician (except for a higher proportion of Indian physicians recommending the vaccine)	Lack of proper guidelines from health authorities regarding HPV vaccine to boys (37.2%) and lack of awareness of availability of the vaccine for boys (32.8%) were the most commonly cited reasons for non-recommendation; majority of physicians believed the first key barriers to offering the vaccine were cost of vaccination (46.6%)
**Yang et al., 2014, South Korea[80]**	Herpes zoster	Survey (details not specified)	400 (35.58%)	a) Physicians<br>b) Adult patients	[+] awareness of recent approval by the Ministry of Food and Drug Safety, routine recommendation of the flu vaccine	Physicians who did not recommend HZ vaccine concerned about costs (90.7%, 78/86) and doubted vaccine effectiveness (58.1%, 50/86); 84.9% (73/86)

						non-recommenders would provide vaccine upon patient request
**Ye et al., 2018, China[65]**	Influenza	33 item questionnaire on perceptions of disease, perceptions of vaccine efficacy and safety, behaviors, awareness, and knowledge	1,340 (97.81%)	a) Physicians, nurses, and interns<br>b) Diabetic adults	[+] believing influenza vaccination would reduce mortality risk of diabetic patients (OR=1.38) & reduce hospitalization costs of diabetic patients (OR=1.43), having received relevant trainings (OR=1.65), self-reported influenza vaccination history (OR=1.35), aware of regional vaccine reimbursement policy (OR=1.62) or of national guideline (OR=6.33), ≥ 10 years of experience (OR: 1.60); [-] working in secondary (OR=0.61) and tertiary hospitals (OR=0.48); HCWs > 50 years old less willing to recommend than < 35 (OR=0.49) and worries about side-effects (OR=0.41); [x] gender, practice setting, belief about risk of influenza and complications, awareness of flu season and access to the vaccine	Awareness that diabetic patients should be treated as an influenza vaccine priority group according to the national influenza vaccination guideline was the most important factor
**Young et al., 2011, US[45]**	HPV	50-item self-administered survey, based on Dillman's Tailored Design Method, on demographics, KAP, and barriers to vaccination	385 (48.7%)	a) Obstetrician-gynecologists and family practitioners in Virginia<br>b) Young women	[+] experience treating HPV-related disease and offering HPV vaccine (OR 2.3);<br>[-] reporting busy staff, inadequate reimbursement, being late adopters of new technology, having safety or efficacy doubts, concerns about compliance with future screening, perceived barriers related to lack of educational materials, inadequate reimbursement with offering the HPV vaccine (OR 0.41) and with recommending the vaccine to patients (OR 0.45);<br>[x] % white patients, safety concerns	94% of respondents state that they are "confident" or "very confident" in the safety and efficacy of the quadrivalent HPV vaccine; 7.3% of family practitioners reported concerns about vaccine efficacy and 5.3% reported concerns about vaccine safety compared to 1.9% and 1.3% of obstetrician-gynecologists, respectively
**Zhang et al., 2012, UK[91]**	Influenza	SDQ w/multi-dimensional Health Locus of Control (MHLC) scales	522 (77.70%)	a) Nurses<br>b) Adult patients	[+] higher knowledge scores and higher risk perception associated with own vaccination and recommendation, being vaccinated themselves, high knowledge scores, higher risk perception; [x] health beliefs scores	Male & female nurses making different vaccination choices may be explained in part by different patterns of health locus of control; respondents w/higher knowledge scores more likely to receive the H1N1 vaccine than those with lower knowledge scores (p = 0.017)
**Zimmerman, 1997, US[46]**	Measles, pertussis, others	15-minute interview using split-panel design questionnaire	1,241 (70%)	a) FP, pediatricians, & GP > 65 years seeing at least moderate numbers of children<br>b) Children > 18 months	[+] more recent year of graduation from medical school, belief side effects do not increase when vaccine is administered during a mild acute illness visit, lower perceived likelihood of parent objection; [-] believing a marked increase in risk when vaccinating a child during a mild acute illness visit, difficulty in determining which causes side effects, decreased efficacy, and parental objection	

**Zimmerman et al., 1998, US[48]**	Measles, pertussis, hepatitis B	15-minute interview using split-panel design questionnaire	1,236 (70%)	a) Physicians seeing at least moderate numbers of children b) Children	[+] physicians more concerned about litigation and higher percentage of vaccinations; [-] those who were highly concerned about litigation; [x] awareness of VICP, ratings of the likelihood of serious adverse effects & referring an insured child to health department for immunization	Among those expressing little concern about litigation, only 12% were unlikely to vaccinate; 86% encouraged vaccination even if a parent was argumentative about possible adverse effects
**Zimmerman, 2002, US[47]**	Varicella	Computer-assisted phone interview with 10 scaled questions	281 (72.7%)	a) PCP from Pennsylvania and Minnesota b) Children 12-18 months, 4-6 years, and 11-12 years	[+] beliefs about likelihood of vaccinated child getting varicella, perception that parents want their children vaccinated, stocking vaccine; [-] belief that serious side effects are not likely, believing vaccine would fail [-] for 12- to 18-month-old and 4- to 6-year-olds; [x] child age, graduation year	Pediatricians who agree with national recommendations and have a positive perception of parental requests are more likely to recommend vaccination.

* Studies listed in alphabetical order by first author's last name; † qualitative studies; ‡ mixed methods.
CME: continuing medical education; FM: family medicine; FP: family physician; GP: general practitioner; HCP: healthcare provider; ID: infectious disease; IM: internal medicine; KAP: knowledge, attitudes, and practices; MMR: Measles, mumps, and rubella; MoH: Ministry of Health; NP: nurse practitioner; PA: physician assistant; PCP: primary care provider; RSV: respiratory syncytial virus; SAQ: self-administered questionnaire; SDQ: self-developed questionnaire; STI: sexually transmitted infections; VICP: vaccine injury compensation program; #-Q: number of questions (in the survey).

Supplementary 3: 14-item Survey Study and Risk of Bias Appraisal (SSRBA)*

Included papers were assessed on each item as Met (M), Partially Met (P), Unmet (U), Unclear (UC), or Not Applicable (N/A)

Author, Publication year (in alphabetical order)	1 Research objectives or hypotheses clearly stated.	2 Sampling method described.	3 Sample randomly selected (or justified if not random).	4 Sample represents target population.	5# Response rate reported.	6# Measures taken to contact or compare non-responders.	7 Adequate sample size.	8 Ethical approval (IRB) obtained.	9 Measures appropriate and clearly described.	10 Methods of analysis described.	11 Effects of variable appropriately specified.	12 Conclusions supported by data (results coherence).	13 Reporting was objective & had adequate details.	14 Study weaknesses and possible bias disclosed.
Allison et al., 2013[76]	M	M	M	M	M	M	M	M	M	M	M	M	M	M
Allison et al., 2016[83]	M	M	P	M	M	UC	M	M	P	M	M	M	M	M
Almughais et al., 2018[97]	M	M	U	M	M	U	M	M	M	M	M	M	M	M
Arnell et al., 2019[120]	M	M	U	M	U	U	M	M	M	M	M	M	M	M
Barnack et al., 2010[119]	M	M	U	U	U†	U	P	M	M	M	U	M	M	M
Bean and Catania, 2013[36]	M	M	U	U	N/A	N/A	P	M	M	M	M	M	M	M
Betscha and Wicker, 2014[126]	M	M	U	UC	M	U	P	U	M	M	M	M	M	M
Böhm et al., 2019[63]	M	M	UC	M	M	M	M	UC	M	M	M	M	M	M
Bovier et al., 2005[94]	M	M	M	M	M	M	M	U	M	M	M	M	M	M
Bruno et al., 2015[90]	M	M	M	M	M	M	P	M	M	M	M	M	M	M
Bynum et al., 2014[66]	M	M	M	UC	M	M	M	M	M	M	M	M	M	M
Canon et al., 2017[131]	M	M	U	UC	M	U	M	M	M	M	M	M	M	M
Collange et al., 2016[54]	M	M	M	M	M	U	M	M	M	M	M	M	M	M
Daley et al., 2006[99]	M	M	M	U	M	M	M	M	M	M	M	M	M	M
Davis et al., 2003[60]	M	M	M	M	M	M	M	M	M	M	M	M	M	M
Dombkowski et al., 2008[117]	M	M	M	P	M	U	M	M	M	M	M	M	M	M
Dubé et al., 2010[92]	M	M	M	M	M	U	M	M	M	M	P	M	M	M
Dubé et al., 2011 (a)[74]	M	M	M	M	M	M	M	M	M	M	M	M	M	M
Dubé et al., 2011 (b)[40]	M	M	M	M	M	M	M	M	M	M	M	M	M	M
Dubé et al., 2011 (c)[39]	M	M	M	M	M	M	M	M	M	M	M	M	M	M
Dubé et al., 2019[55]	M	M	UC	U	U	U	M	M	M	M	M	M	M	M
Duval et al., 2009[41]	M	M	M	U	M	M	M	M	M	M	M	M	M	M
Ehresmann et al., 2000[84]	M	M	M	M	M	M	M	UC	P	M	M	M	M	P
Esposito et al., 2007[81]	M	M	P	M	M	U	M	M	M	M	M	M	M	M
Fagnan et al., 2011[116]	M	M	M	M	M	M	M	M	M	M	M	M	M	M
Farias et al., 2017[61]	M	M	M	P	M	U	P	M	M	M	M	M	M	M
Feemster et al., 2008[77]	M	M	P	U	M	U	P	UC	M	M	M	M	M	M
Ferrara et al., 2018[93]	M	M	M	U	M	M	M	M	M	M	M	M	M	M
Flicoteaux et al., 2014[89]	M	M	U	P	M	U	M	M	M	M	M	M	M	M
Gesser-Edelsburg et al., 2017[88]	M	M	U	M	P	U	P	M	M	M	M	M	M	M
Gilca et al., 2009[57]	M	M	M	M	M	M	M	M	M	M	M	M	M	M
Glavier et al., 2019[82]	M	M	M	M	M	U	M	M	M	M	M	M	M	M
Gust et al., 2008[118]	M	M	M	U	M	U	M	UC	M	M	M	M	M	M
Hobeika et al., 2019[95]	M	M	M	U	M	U	P	UC	M	M	M	M	M	M
Hofstetter et al., 2017[103]	M	M	M	P	M	M	M	M	M	M	M	M	M	M
Hopkins et al., 2009[124]	M	M	U	U	M	M	M	UC	M	P	P	M	M	M
Hoque et al., 2016[121]	M	U	UC‡	U	U	U	M	M	U	U	M	M	M	M
Hurley et al., 2008[85]	M	M	M	M	M	M	M	M	M	M	M	M	M	M
Inoue et al., 2011[112]	M	M	M	P	M	U	M	M	M	M	M	M	M	M
Jaoude et al., 2018[101]	M	M	U	P	M	M	M	M	M	M	M	M	M	M
Jaramillo et al., 2009[73]	M	M	M	UC	M	U	M	M	M	M	M	M	M	M
Kahn et al., 2005[49]	M	M	M	P	M	M	M	M	M	M	M	M	P	M
Kao et al., 2012[122]	M	M	UC	M	M	M	M	M	M	M	M	M	M	M
Karlsson et al., 2019[58]	M	M	M	M	M	UC	M	M	M	M	M	M	M	M
Kassianos et al., 2018[51]	M	M	U	P	U	U	M	UC	P	M	P	M	P	M

Kempe et al., 2007[42]	M	M	M	M	M	M	M	M	M	M	M	M	M	M
Kempe et al., 2018[104]	M	M	M	M	M	M	M	M	M	M	M	M	M	M
Klett-Tammen et al., 2016[53]	M	M	M	M	M	U	M	M	P	M	P	M	P	M
Krishnaswamy et al., 2019[114]	M	M	P	P	M	P	M	M	M	M	M	M	M	M
Lehmann et al., 2017[75]	M	M	M	U	M	M	M	N/A§	M	M	M	M	M	M
LeMaréchal et al., 2017[79]	M	M	P	M	M	U	M	M	M	M	M	M	M	M
LeMaréchal et al., 2018[105]	M	M	P	P	M	U	M	M	P	M	P	M	P	M
Lutringer-Magnin et al., 2011[37]	M	M	P	P	U	U	M	M	P	M	P	P	P	P
MacDougall et al., 2015[38]	M	M	M	M	M	U	M	M	P	M	P	M	M	P
Massin et al., 2015[43]	M	M	P	P	M	U	M	M	M	M	P	M	P	M
Mui et al., 2013[67]	M	M	M	M	M	M	M	M	M	M	M	P	P	U
Napolitano et al., 2018[72]	M	M	M	M	M	M	M	M	M	M	M	M	M	M
Neufeind et al., 2020[106]	M	M	M	M	M	P	M	M	M	M	M	M	M	M
Newman and Taylor, 1998[87]	M	M	M	M	M	M	M	UC	M	M	M	M	M	M
Nikolic et al., 2015[78]	M	M	P	P	M	P	P	UC	M	M	M	M	P	M
Noh et al., 2016[132]	M	P	U	P	M	M	M	M	P	M	M	M	M	M
Pavia et al., 2003[125]	M	U	M	M	M	M	P	UC	M	M	M	P	P	P
Pelullo et al., 2020[96]	M	M	M	M	M	M	M	M	M	M	M	M	M	M
Perkins and Clark, 2012[35]	M	M	U	P	N/A	N/A	M	M	M	M	M	M	M	M
Power et al., 2009[98]	M	M	P	P	M	M	M	M	M	M	P	P	P	M
Praphasiri et al., 2017[68]	M	M	P	P	M	M	M	M	M	M	M	M	M	M
Raude et al., 2016[62]	M	M	M	M	M	M	M	M	M	M	M	M	M	M
Riedesel et al., 2005[50]	M	M	M	M	M	M	P	M	M	M	M	M	M	M
Rutten et al., 2017[123]	M	M	M	M	M	NC	M	M	M	M	M	M	M	M
Shibli et al., 2017[113]	M	M	M	U	M	U	M	M	M	M	M	M	M	M
Shibli et al., 2019[107]	M	M	U	P	M	U	M	M	M	M	M	M	M	M
Soon et al., 2015[127]	M	M	U	U	M	M	U	M	M	M	P	M	M	M
Stefanoff et al., 2020[108]	M	M	M	M	M	P	M	M	M	M	M	M	M	M
Suryadevara et al., 2015[69]	M	M	P	P	M	U	M	M	M	M	M	M	M	M
Taylor et al., 2014[70]	M	M	M	M	M	M	M	M	M	M	M	M	M	M
Tong et al., 2008[111]	M	M	M	P	M	U	M	M	M	M	M	M	M	M
Topuridze et al., 2010[129]	M	M	M	M	M	U	M	M	M	M	M	M	M	M
Torun and Torun, 2010[56]	M	M	U	U	M	U	M	UC	M	M	M	M	M	M
Tuckerman et al., 2020[71]	M	M	U	M	M	M	M	M	M	M	M	M	M	M
Vadaparampil et al., 2013[109]	M	M	M	M	M	M	M	M	M	M	M	P	P	M
Vadaparampil et al., 2016[52]	M	M	P	P	M	M	M	UC	M	M	M	M	M	M
Verger et al., 2015[64]	M	M	M	M	M	M	M	M	M	M	M	M	M	M
Verger et al., 2016[110]	M	M	M	M	M	M	M	M	M	M	M	M	M	M
Vezzosi et al., 2019[44]	M	M	M	U	M	P	U	UC	P	P	P	P	P	P
Vilca et al., 2018[128]	M	M	U	M	M	UC	M	M	P	M	P	M	M	M
Wagner et al., 2015[130]	M	M	M	P	M	M	M	UC	M	M	P	M	M	M
Wilcox et al., 2019[86]	M	M	U	U	M	M	M	M	M	M	M	M	M	M
Wilcox et al., 2020[115]	M	M	M	M	M	M	M	M	M	M	M	M	M	M
Wong et al., 2017[100]	M	M	M	M	M	M	M	M	M	M	P	M	M	M
Yang et al., 2014[80]	M	U	U	U	M	U	M	M	U	M	U	P	P	M
Ye et al., 2018[65]	M	M	M	M	M	M	M	M	M	M	M	M	M	M
Young et al., 2011[45]	M	M	M	M	M	U	M	M	P	M	P	P	M	M
Zhang et al., 2012[91]	M	M	U	U	M	U	M	M	M	M	M	M	M	M
Zimmerman, 1997[46]	M	M	M	M	M	U	M	UC	P	M	P	M	P	M
Zimmerman et al., 1998[48]	M	M	M	M	M	U	M	UC	P	M	M	M	P	M
Zimmerman, 2002[47]	M	M	U	P	M	U	M	M	M	M	M	M	M	M

*The SSRBA scale was developed by the research team, adapted Circum Network's six-survey-building-block framework (questionnaire, sampling, data collection/management/analysis, and reporting) **[30]**, BETs' critical appraisals for surveys and qualitative studies **[31]**,**[32]**, and Joanna Briggs Institute's checklist for systematic reviews **[33]**.

# Items 5 and 6 are for quantitative studies only; qualitative studies are marked as N/A.

† The research team stopped collecting responses after obtained required number.

‡ Methods was only reported in an earlier publication as part of the same study.

§ The study authors indicated ethics approval was not required according to the Medical Research Involving Human Subjects Act in the Netherlands.
